# Joint Effects of Prenatal Antibiotics, Mode of Birth and Breastfeeding Duration on Childhood Infections: The Norwegian MoBa Cohort Study

**DOI:** 10.1111/ppe.70120

**Published:** 2026-02-23

**Authors:** Isobel M. F. Todd, Lars Henning Pedersen, Jessica E. Miller, David P. Burgner, Maria C. Magnus

**Affiliations:** ^1^ Murdoch Children's Research Institute Parkville Victoria Australia; ^2^ Department of Paediatrics The University of Melbourne Parkville Victoria Australia; ^3^ Clinical Medicine Aarhus University Aarhus Denmark; ^4^ Department of Obstetrics and Gynecology Aarhus University Hospital Aarhus Denmark; ^5^ Department of General Medicine, Infectious Diseases Royal Children's Hospital Melbourne Parkville Victoria Australia; ^6^ Faculty of Health, Deakin University Victoria Australia; ^7^ Centre for Fertility and Health Norwegian Institute of Public Health Oslo Norway

**Keywords:** breastfeeding, caesarean, childhood, infections, prenatal antibiotics

## Abstract

**Background:**

Prenatal antibiotic exposure and caesarean births are associated with an increased risk of hospitalised infection in children, but few studies have evaluated their impact on less severe infections and possible joint effects. Conversely, longer breastfeeding duration is protective against infections, but whether this effect varies according to previous perinatal exposures has not been explored. These three exposures are all hypothesised to influence the infant microbiome.

**Objectives:**

To examine the individual and joint effects of prenatal antibiotic exposure, caesarean birth, and breastfeeding duration for both the incidence of infections and for hospitalisation for infection up to age three.

**Methods:**

Participants were from the Norwegian Mother, Father, and Child Cohort Study (MoBa). The three exposures were prenatal antibiotic exposure, caesarean birth, and breastfeeding duration. Using quasi‐Poisson regression, we analysed the number of infections and hospitalisation for infections according to: (1) each exposure individually, (2) combined exposure to prenatal antibiotics and caesarean birth and (3) breastfeeding duration across the strata of prenatal antibiotics and birth mode.

**Results:**

Among 45,485 children followed from birth to age three, there was weak evidence for an increased number of infections according to the exposures of interest (incidence rate ratio for prenatal antibiotics 1.04, 95% CI 1.02, 1.05; caesarean birth 1.02, 95% CI 1.00, 1.03; breastfeeding < 6 months 1.04, 95% CI 1.02, 1.05), while there was an increased risk of hospitalised infection (risk ratio for prenatal antibiotics 1.11, 95% CI 1.05, 1.17; caesarean birth 1.20, 95% CI 1.14, 1.27; breastfeeding < 6 months 1.16, 95% CI 1.10, 1.22). Analyses of combined effects showed a 1.55‐fold (95% CI 1.24, 1.93) increased risk of hospitalisation for infection with all three exposures compared to none.

**Conclusions:**

Prenatal antibiotic exposure, caesarean birth and shorter breastfeeding duration were each associated with an increased risk of hospitalisation for infection in early childhood, with higher magnitude when multiple exposures occurred.

## 1 Background

Childhood infections are a leading cause of hospitalisation and result in a substantial direct and indirect health and economic burden [1, 2, 3]. Previous population‐based registry studies have found that prenatal antibiotic exposure, caesarean birth, and shorter breastfeeding duration are associated with an increased risk of hospitalised infections in children [4, 5, 6, 7, 8, 9, 10, 11, 12]. Smaller cohort studies show inconclusive findings on whether prenatal antibiotic exposure and caesarean birth are also associated with an increased incidence of non‐hospitalised infections [13, 14, 15, 16, 17, 18]. There are few studies in large cohorts that compare the risk associated with these exposures according to the number and severity of infections. This is important to explore given the significant burden caused by non‐hospitalised infections in addition to hospitalised infections.

A proposed mechanism by which prenatal antibiotic exposure and caesarean birth influence infection risk is through their effects on the infant microbiome and subsequent impacts on immune development and responses [19, 20, 21, 22]. The maternal microbiome is disrupted by antibiotic use, and the child may acquire this dysbiosis during delivery [20]. Caesarean birth results in a lack of exposure to the maternal enteric microbiota, which may similarly disrupt the seeding of the infant's microbiome [20]. Conversely, breast milk contains many beneficial biological components and is protective against early‐life infections [9, 23]. Few studies have considered the possible joint effects of these key exposures in the context of childhood infections.

We therefore aimed to investigate the individual and joint effects of these exposures on hospitalised and non‐hospitalised infections in the first 36 months of life. We hypothesised that the beneficial effects of longer breastfeeding duration may be more evident in children exposed to antibiotics prenatally or born by caesarean.

## 2 Methods

### 2.1 Study Population

We studied participants in the Norwegian Mother, Father, and Child Cohort Study (MoBa), a population‐based national birth cohort study that recruited pregnant women and their partners at approximately 17 weeks' gestation between 1999 and 2008 in Norway. The participation rate was 41% and over 100,000 mother–child pairs participated [24, 25]. All participants provided written informed consent. Further details on the cohort have been described previously [24, 25]. Information was obtained through questionnaires administered during pregnancy and at regular intervals post‐partum. Additional information was obtained through linkage to the Medical Birth Registry of Norway (MBRN).

**FIGURE 1:**
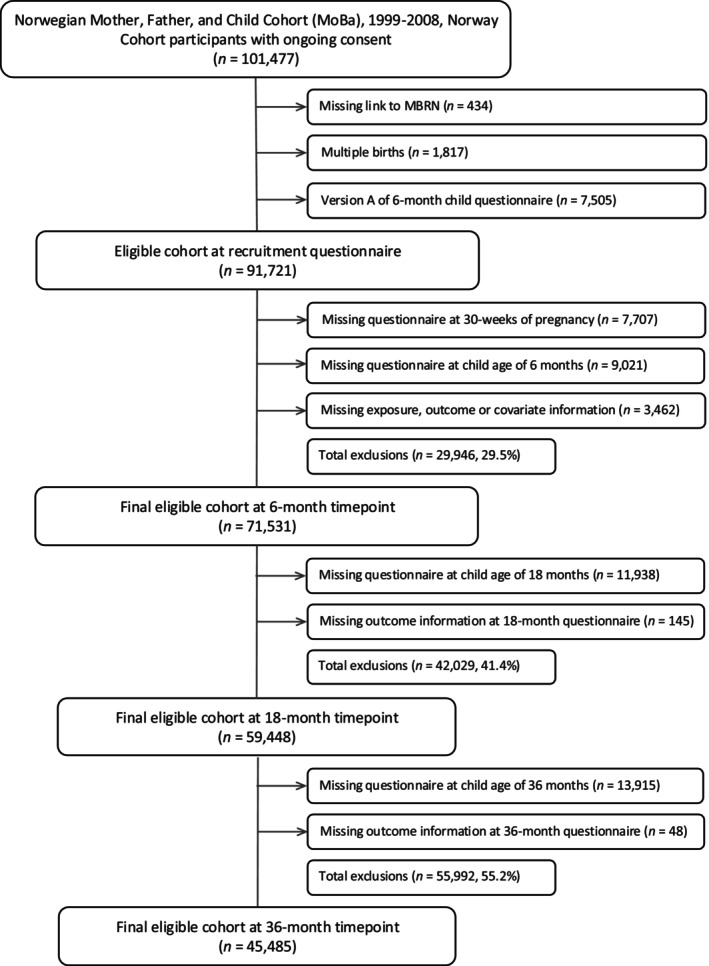
Study population selection from the Norwegian Mother, Father, and Child Cohort study.

There were 101,477 mother–child pairs with ongoing consent and information from the recruitment questionnaire in the available research data files. We excluded children without a link to the MBRN (*n* = 434), multiple births (*n* = 1817 births), and those administered the first version of the 6‐month questionnaire (version A) (*n* = 7505), as this version did not ask about the number of infections. This resulted in a total of 91,721 eligible mother–child pairs with information at the recruitment questionnaire (17 weeks' gestation). This study used information from five MoBa questionnaires, including at 17 and 30 weeks' gestation and at 6, 18, and 36 months of age. From these, the final sample included 71,531, 59,448, and 45,485 children in the analyses of outcomes at 6, 18, and 36 months, respectively (Figure 1).

### 2.2 Exposures

The exposures of interest were prenatal antibiotic exposure, mode of birth, and breastfeeding duration. Prenatal antibiotic exposure was determined from MoBa questionnaires at 17 and 30 weeks' gestation and 6 months post‐partum. Mothers were asked if they had experienced a list of specific illnesses/health problems during pregnancy and for each illness/health problem if they had taken any medication. Maternal‐reported medications were coded by MoBa personnel to the Anatomical Therapeutic Chemical (ATC) classification. The prenatal antibiotic exposed group were those with any reported medication use during pregnancy with an ATC code starting with ‘J01’ (antibacterials for systemic use), and the reference group those without. Mode of birth was determined from the MBRN, with caesarean birth defined as the exposed group and vaginal birth as the reference group. Breastfeeding duration was determined from the questionnaires at 6‐ and 18‐months child age. We focused on duration of any breastfeeding (rather than exclusive breastfeeding) and categorised this as less than 6 months (exposed) versus 6 months or longer (reference). This exposure was chosen to align both with our hypothesis that any breastmilk exposure would be delivering beneficial components and with previous findings from the MoBa cohort regarding breastfeeding and infections [12].

### 2.3 Outcomes

The primary outcomes in this study were childhood infections in the first 6, 18, and 36 months of life specifically: (1) the number of infection episodes (irrespective of severity) and (2) hospitalisation for an infection. The infection types as listed in the questionnaires were: (1) common cold, (2) throat infection, (3) ear infection, (4) pseudocroup, (5) bronchitis/respiratory syncytial virus/pneumonia, (6) gastric flu/diarrhoea, (7) urinary tract infection, (8) febrile convulsions, and (9) chickenpox. For each listed infection type, parents were asked: whether the child had experienced the infection (yes/no); the number of times the child experienced the infection; whether they had attended the GP; whether they had been admitted to hospital. Our primary outcomes were measured as the total number of infection episodes reported across all infection types between birth and each timepoint and a binary outcome of whether the child had been admitted to hospital for any infection by each timepoint. Secondary outcomes included: (1) the same measures but limited to respiratory infections (infection types 1–5 above) and (2) the number of infection episodes excluding common colds (infection types 2–9 above).

### 2.4 Covariates

**FIGURE 2:**
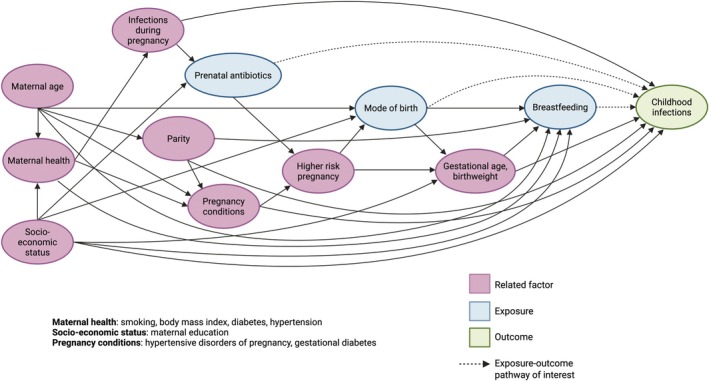
Causal directed acyclic graph.

We identified maternal and offspring background characteristics related to the exposures and outcomes from previous literature and constructed a hypothesised causal model through a directed acyclic graph (DAG) [26] (Figure 2). Maternal characteristics included highest obtained educational level, infection during pregnancy, pre‐pregnancy body mass index (BMI), smoking, and diabetes, primarily obtained through MoBa questionnaires but supplemented with MBRN information. Pregnancy and child characteristics identified from the MBRN included maternal age, hypertension (pre‐pregnancy or pregnancy‐induced), pre‐eclampsia (including pre‐eclampsia, eclampsia, and HELLP), parity, gestational age, birth weight, and birth year. Covariates were categorised as shown in Table 1. Descriptive statistics were compared in children who were and weren't lost to follow‐up between the 6‐month and 36‐month questionnaires.

**TABLE 1:** Descriptive statistics by exposures for study population analysed at 36‐month timepoint.

	Prenatal antibiotics		Mode of birth		Breastfeeding	
	No	Yes	Vaginal	Caesarean	≥ 6 months	< 6 months
	39,900 (88.7%)	5585 (12.3%)	39,360 (86.5%)	6125 (13.5%)	38,706 (85.1%)	6779 (14.9%)
**Maternal and pregnancy factors**						
Maternal age, years						
< 20	172 (0.4)	18 (0.3)	166 (0.4)	24 (0.4)	106 (0.3)	84 (1.2)
20–24	3209 (8.0)	457 (8.2)	3255 (8.3)	411 (6.7)	2680 (6.9)	986 (14.5)
25–29	13,333 (33.4)	1826 (32.7)	13,404 (34.1)	1755 (28.7)	12,824 (33.1)	2335 (34.4)
30–34	15,976 (40.0)	2325 (41.6)	15,843 (40.3)	2458 (40.1)	15,998 (41.3)	2303 (34.0)
35+	7210 (18.1)	959 (17.2)	6692 (17.0)	1477 (24.1)	7098 (18.3)	1071 (15.8)
Maternal education						
1–2‐year high school	1338 (3.4)	157 (2.8)	1244 (3.2)	251 (4.1)	1010 (2.6)	485 (7.2)
3‐year high school	4309 (10.8)	553 (9.9)	4094 (10.4)	768 (12.5)	3802 (9.8)	1060 (15.6)
9‐year high school	468 (1.2)	63 (1.1)	438 (1.1)	93 (1.5)	339 (0.9)	192 (2.8)
Vocational high school	4408 (11.0)	607 (10.9)	4213 (10.7)	802 (13.1)	3611 (9.3)	1404 (20.7)
Bachelor's degree	17,928 (44.9)	2617 (46.9)	17,988 (45.7)	2557 (41.7)	17,911 (46.3)	2634 (38.9)
Master's degree or higher	11,449 (28.7)	1588 (28.4)	11,383 (28.9)	1654 (27.0)	12,033 (31.1)	1004 (14.8)
Parity = nulliparous, *n* (%)	19,708 (49.4)	2434 (43.6)	18,718 (47.6)	3424 (55.9)	18,418 (47.6)	3724 (54.9)
BMI before pregnancy						
< 18.5	1147 (2.9)	156 (2.8)	1171 (3.0)	132 (2.2)	1093 (2.8)	210 (3.1)
18.5–24.9	26,775 (67.1)	3617 (64.8)	26,911 (68.4)	3481 (56.8)	26,801 (69.2)	3591 (53.0)
25–29.9	8515 (21.3)	1224 (21.9)	8156 (20.7)	1583 (25.8)	7955 (20.6)	1784 (26.3)
30+	3463 (8.7)	588 (10.5)	3122 (7.9)	929 (15.2)	2857 (7.4)	1194 (17.6)
Smoking						
Never smoker	21,574 (54.1)	3125 (56.0)	21,625 (54.9)	3074 (50.2)	21,827 (56.4)	2872 (42.4)
Former smoker	8564 (21.5)	1136 (20.3)	8385 (21.3)	1315 (21.5)	8419 (21.8)	1281 (18.9)
Before pregnancy	7109 (17.8)	978 (17.5)	6854 (17.4)	1233 (20.1)	6517 (16.8)	1570 (23.2)
Early pregnancy	507 (1.3)	66 (1.2)	463 (1.2)	110 (1.8)	423 (1.1)	150 (2.2)
During pregnancy	2146 (5.4)	280 (5.0)	2033 (5.2)	393 (6.4)	1520 (3.9)	906 (13.4)
Diabetes						
No diabetes	39,336 (98.6)	5474 (98.0)	38,887 (98.8)	5923 (96.7)	38,193 (98.7)	6617 (97.6)
Pre‐gestational diabetes	248 (0.6)	41 (0.7)	189 (0.5)	100 (1.6)	215 (0.6)	74 (1.1)
Gestational diabetes	316 (0.8)	70 (1.3)	284 (0.7)	102 (1.7)	298 (0.8)	88 (1.3)
Hypertension, *n* (%)	1007 (2.5)	153 (2.7)	928 (2.4)	232 (3.8)	940 (2.4)	220 (3.2)
Pre‐eclampsia/eclampsia/HELLP, *n* (%)	1474 (3.7)	220 (3.9)	1127 (2.9)	567 (9.3)	1315 (3.4)	379 (5.6)
Infection during pregnancy = yes, *n* (%)	30,485 (76.4)	5434 (97.3)	31,115 (79.1)	4804 (78.4)	30,651 (79.2)	5268 (77.7)
Year of birth						
2002	2512 (6.3)	352 (6.3)	2504 (6.4)	360 (5.9)	2404 (6.2)	460 (6.8)
2003	5439 (13.6)	832 (14.9)	5457 (13.9)	814 (13.3)	5256 (13.6)	1015 (15.0)
2004	5735 (14.4)	828 (14.8)	5693 (14.5)	870 (14.2)	5496 (14.2)	1067 (15.7)
2005	6387 (16.0)	916 (16.4)	6323 (16.1)	980 (16.0)	6224 (16.1)	1079 (15.9)
2006	6932 (17.4)	905 (16.2)	6734 (17.1)	1103 (18.0)	6702 (17.3)	1135 (16.7)
2007	6221 (15.6)	874 (15.6)	6108 (15.5)	987 (16.1)	6078 (15.7)	1017 (15.0)
2008	5290 (13.3)	706 (12.6)	5182 (13.2)	814 (13.3)	5170 (13.4)	826 (12.2)
2009	1384 (3.5)	172 (3.1)	1359 (3.5)	197 (3.2)	1376 (3.6)	180 (2.7)
**Child factors**						
Child sex = male, *n* (%)	20,314 (50.9)	2843 (50.9)	19,894 (50.5)	3263 (53.3)	19,569 (50.6)	3588 (52.9)
Gestational age (weeks)						
< 32	149 (0.4)	16 (0.3)	54 (0.1)	111 (1.8)	114 (0.3)	51 (0.8)
32–33	240 (0.6)	40 (0.7)	150 (0.4)	130 (2.1)	208 (0.5)	72 (1.1)
34–35	622 (1.6)	89 (1.6)	476 (1.2)	235 (3.8)	531 (1.4)	180 (2.7)
36–37	2494 (6.3)	346 (6.2)	2249 (5.7)	591 (9.6)	2343 (6.1)	497 (7.3)
38–40	24,930 (62.5)	3555 (63.7)	24,950 (63.4)	3535 (57.7)	24,324 (62.8)	4161 (61.4)
> 40	11,465 (28.7)	1539 (27.6)	11,481 (29.2)	1523 (24.9)	11,186 (28.9)	1818 (26.8)
Birth weight						
< 2500	1004 (2.5)	147 (2.6)	657 (1.7)	494 (8.1)	865 (2.2)	286 (4.2)
2500–2999	3436 (8.6)	475 (8.5)	3270 (8.3)	641 (10.5)	3238 (8.4)	673 (9.9)
3000–3499	11,693 (29.3)	1602 (28.7)	11,745 (29.8)	1550 (25.3)	11,265 (29.1)	2030 (29.9)
3500–4000	15,350 (38.5)	2131 (38.2)	15,464 (39.3)	2017 (32.9)	15,055 (38.9)	2426 (35.8)
> 4000	8417 (21.1)	1230 (22.0)	8224 (20.9)	1423 (23.2)	8283 (21.4)	1364 (20.1)
**Exposures of interest**						
Prenatal antibiotics = yes	NA	NA	4809 (12.2)	776 (12.7)	4700 (12.1)	885 (13.1)
Caesarean birth	5349 (13.4)	776 (13.9)	NA	NA	4802 (12.4)	1323 (19.5)
Breastfeeding < 6 months	5894 (14.8)	885 (15.8)	5456 (13.9)	1323 (21.6)	NA	NA

### 2.5 Statistical Analysis

We first analysed the association between each exposure individually for the two infection outcomes and three timepoints using multivariable quasi‐Poisson regression models to analyse the number of infections (reporting incidence rate ratios [IRR]) and to analyse the risk of hospitalisation (reporting risk ratios [RR]) [27]. Pre‐specified confounders identified from our DAG included maternal age, BMI, smoking, education, diabetes, parity, and birth year for all three exposures. Prenatal antibiotics and mode of birth models also adjusted for infections during pregnancy. Mode of birth models additionally adjusted for prenatal antibiotics, hypertension, and pre‐eclampsia, while breastfeeding models additionally adjusted for mode of birth, birth weight, and gestational age. Robust variance estimates with clustering for maternal ID were calculated using the ‘lmtest’ R package [28] to account for non‐independence of siblings.

Second, we analysed the interaction between prenatal antibiotic exposure and mode of birth. For this, we calculated effect estimates for each combination of the two binary exposures with those unexposed to prenatal antibiotics and born by vaginal birth as the reference category. We report results evaluating both the presence of multiplicative interaction (RR_11_/RR_01_RR_10_) and additive interaction (relative excess risk due to interaction [RERI]; RR_11_—RR_01_—RR_10_ + 1), where RR represents the effect measure (e.g., risk ratio or incidence rate ratio) and the subscripts denote exposure status (0 = unexposed, 1 = exposed) [29]. Third, we assessed whether the combination of prenatal antibiotics and mode of birth acted as an effect modifier for breastfeeding duration. We calculated effect estimates for each combination of the three binary exposures, with those unexposed to prenatal antibiotics, born by vaginal birth and breastfed for 6 or more months as the reference category. We also calculated the effect estimates for breastfeeding duration less than 6 months compared to more than 6 months in each stratum of prenatal antibiotics and mode of birth as a combined variable (i.e., four strata total) to assess effect modification. All analyses were conducted using R statistical software version 4.2.3 [30]. Interaction measures were calculated using the interactionR package with the default parameters [31].

In sensitivity analyses, we repeated the analyses for the sample who were followed to 36 months but calculated separate estimates for the 0–6‐month, 6–18‐month, and 18–36‐month age windows to evaluate whether the exposures of interest had a greater influence on early infections. We also conducted analyses where exclusive breastfeeding less than 6 months versus 6 or more months was used as the breastfeeding exposure variable and analyses for emergency and elective caesarean separately.

To account for potential selection bias from participant loss to follow‐up across successive questionnaires, we conducted weighted analyses and compared these with our unweighted complete‐case analyses. Inverse probability weights based on the probability of being included in the analysis at each timepoint were generated using the eligible sample at recruitment (*n* = 91,721). Estimates from the two analyses were similar and therefore, estimates from the weighted analysis are presented throughout. Table S1 shows a comparison of the unweighted and weighted analyses.

### 2.6 Missing Data

From the 74,993 who responded to the 17‐, 30‐week and 6‐month questionnaires, we excluded those with missing data required to define exposure, outcome and confounder variables. This included variables for breastfeeding (*n* = 101 missing), infections (*n* = 266 at 6 months), maternal BMI (*n* = 1282), maternal smoking (*n* = 413), maternal education (*n* = 1461) and gestational age (*n* = 9). Identical exclusion processes were applied for the samples at 18 and 36 months (Figure 1). This resulted in 4.5%–4.6% of participants excluded overall at the three timepoints due to missing data (Figure 1).

### 2.7 Ethics

The establishment of MoBa and initial data collection was based on a licence from the Norwegian Data Protection Agency and approval from The Regional Committees for Medical and Health Research Ethics. The MoBa cohort is currently regulated by the Norwegian Health Registry Act. The current study was approved by the Regional Committee for Medical and Health Research Ethics of South/East Norway (Ref. 2018/24492/REK sør‐øst).

## 3 Results

A total of 12.3% children were exposed to prenatal antibiotics, 13.5% to caesarean birth, and 14.9% to breastfeeding less than 6 months among 45,485 children followed to 36 months age. The proportion with breastfeeding duration less than 6 months was slightly higher among those with follow‐up information at 6 and 18 months (16.5% at 6 months, 15.6% at 18 months). The distribution of maternal and child characteristics for each exposure is shown in Table 1 and Tables S2, S3 for the three timepoints. Some notable differences were higher maternal age, BMI, pre‐eclampsia, preterm birth, and low birthweight among caesarean births and higher BMI, higher smoking during pregnancy, and a lower proportion with university education among those breastfed for less than 6 months. When comparing the characteristics of those who were and weren't lost to follow‐up between the 6‐ and 36‐month questionnaires, mothers lost to follow‐up were on average younger, had lower education levels, higher smoking rates, and a higher proportion with breastfeeding for less than 6 months (Table S4).

**FIGURE 3:**
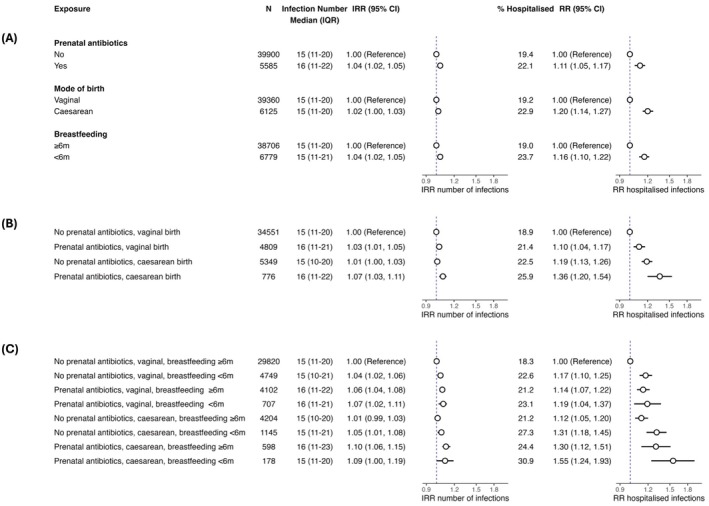
Associations of exposures with number of infections and hospitalisation for infection from birth to 36 months age. (A) Associations for individual exposures. (B) Associations for combinations of prenatal antibiotic exposure and mode of birth. (C) Association for combinations of all three exposures.

Among all children, the median number of infections was 1 (interquartile range [IQR] 1–2) in the first 6 months of life, 8 (IQR 5–11) in the first 18 months, and 15 (IQR 11–20) in the first 36 months. IRR estimates for the number of infections from birth to 36 months of age for each exposure ranged from 1.02 (95% CI 1.00–1.03) for caesarean birth to 1.04 (95% CI 1.02–1.05) for both prenatal antibiotic exposure and breastfeeding less than 6 months (Figure 3). 4%, 9%, and 20% of children were hospitalised for an infection during the first 6, 18, and 36 months of life respectively. Increased risks of hospitalisation for infection were observed following prenatal antibiotic exposure, caesarean birth, and breastfeeding duration less than 6 months, with adjusted estimates from birth to 36 months of age ranging from 1.11 (95% CI 1.05–1.17) for prenatal antibiotic exposure to 1.20 (95% CI 1.14–1.27) for caesarean birth.

The associations between prenatal antibiotic exposure and caesarean birth with the number of infections and with the risk of hospitalised infections in interaction analyses showed the same pattern as for the individual analyses of these exposures (Figure 3). A stronger association was seen when both exposures were present, compared to either exposure on its own (IRR for number of infections: 1.07, 95% CI 1.03–1.11; RR for hospitalisation for infection: 1.36, 95% CI 1.20–1.54 for analyses up to age 36 months) (Figure 3). However, we did not observe evidence of interaction between exposures; the multiplicative interaction terms were 1.02 (95% CI 0.98–1.07) and 1.03 (95% CI 0.90–1.20), and the RERI terms were 0.025 (95% CI −0.018 to 0.067) and 0.066 (95% CI −0.118 to 0.250) for analyses of number of infections and hospitalisation for infection respectively. This indicates no added risk posed by the combined exposure beyond that expected from their individual effects.

In analyses including combinations of all three exposures, estimates ranged from a null association to a 1.10‐fold higher incidence rate of infections across the exposure combinations (Figure 3). For hospitalisation for infection, a pattern of increasing risk for greater numbers of adverse exposures was observed, with up to a 1.55‐times higher risk (95% CI 1.24–1.93) among children with all three exposures observed when evaluating hospitalisation up to 36 months of age (Figure 3). There was no clear evidence of differences across strata when comparing whether the association between shorter breastfeeding duration and infection outcomes differed according to the strata of prenatal antibiotic exposure and mode of birth (Table S5).

**FIGURE 4:**
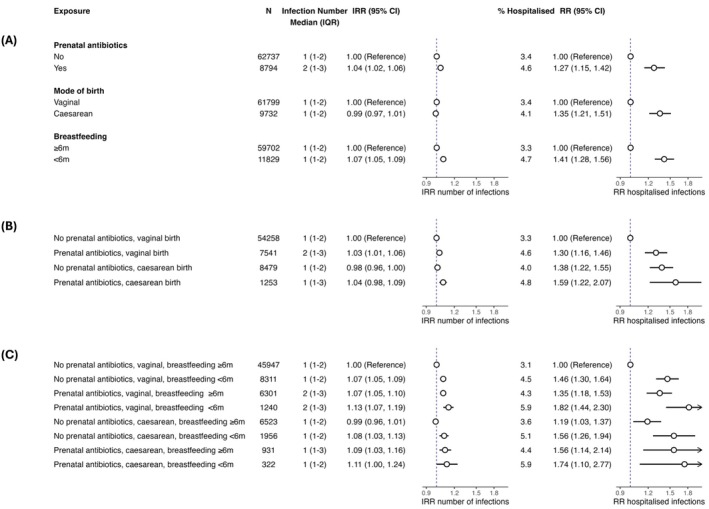
Associations of exposures with number of infections and hospitalisation for infection from birth to 6 months age. (A) Associations for individual exposures. (B) Associations for combinations of prenatal antibiotic exposure and mode of birth. (C) Association for combinations of all three exposures.

**FIGURE 5:**
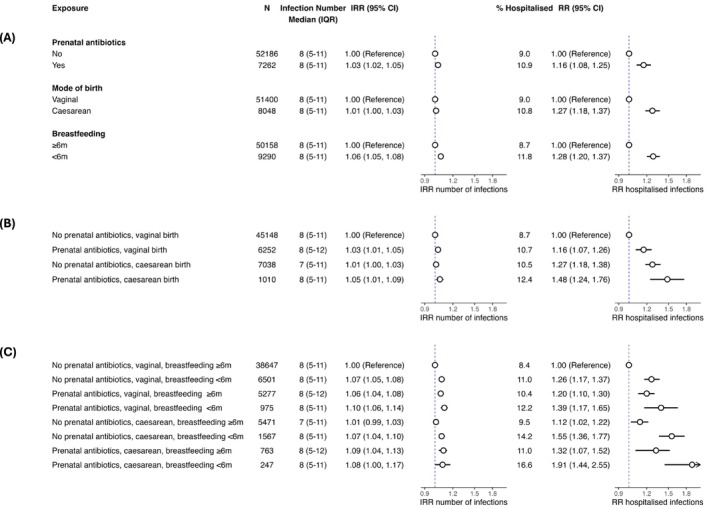
Associations of exposures with number of infections and hospitalisation for infection from birth to 18 months age. (A) Associations for individual exposures. (B) Associations for combinations of prenatal antibiotic exposure and mode of birth. (C) Association for combinations of all three exposures.

In analyses considering infections from 0 to 6 months (*n* = 71,531) and 0 to 18 months (*n* = 59,448) age in the larger available samples, similar patterns were observed as in our main analyses with higher magnitudes of association for shorter age of follow‐up (Figures 4 and 5).

Similar results to the main analyses were observed when evaluating respiratory infections specifically (Figure S1). When considering the total number of infections excluding common colds, the observed associations were slightly higher in magnitude (Figure S2).

When we evaluated the timing of infections for each individual exposure, differences in the incidence rate of infections by age window were very small, while the risk of hospitalisation for infection was present for each age window but higher in magnitude closer to birth (Figure S3).

Similar patterns to our main analyses were observed when considering exclusive breastfeeding rather than any breastfeeding as our exposure variable (Figure S4). When emergency and elective caesarean were considered separately, associations were similar for each caesarean type with overlapping confidence intervals (Figure S5).

## 4 Comment

### 4.1 Principal Findings

This study investigated the individual and joint associations of prenatal antibiotic exposure, mode of birth, and breastfeeding duration with the incidence of infection and hospitalisation for infection in early childhood. In the first 36 months of life, we observed null to modest increased rates by 1%–10% in the total number of infections for the individual and joint effects of the three exposures. We found an increased risk of hospitalisation for infection associated with each of the three exposures ranging between 11% to 20% for the individual exposures from birth to 36 months of age. The magnitude of risk increased further according to the number of adverse exposures in analyses of joint effects. However, we did not find evidence of interaction between prenatal antibiotic exposure and caesarean birth. Similarly, we did not observe evidence supporting the hypothesis that the effect of shorter breastfeeding duration on early childhood infections differed among prenatal antibiotic exposure and mode of birth strata.

### 4.2 Strengths of the Study

This is the first study to examine the joint effects of these exposures for both non‐hospitalised and hospitalised childhood infections, despite hypotheses that their relationships with infection may involve similar mechanistic pathways [4, 5, 6, 7, 14, 23]. The cohort analysed was much larger than previous studies of non‐hospitalised infections and we were able to contrast less severe infections with hospitalised infections. The population‐based recruitment of the cohort enhances the generalisability of our findings, while the detailed information from the questionnaires and linked registry data allowed for adjustment of a broad range of potential confounders.

### 4.3 Limitations of the Data

Several study designs and contextual considerations are important when interpreting these findings. First, measurement error from self‐reported data may impact our findings, particularly for the outcome of the number of infections. This was ascertained at three questionnaires, with an 18‐month gap between the final two questionnaires. There is likely to be variation in how parents define an infection episode and their recall of the number of infection episodes. Although this is unlikely to differ by exposure status, this may have biased our results towards the null. We did observe slightly higher associations in analyses excluding common colds, which may be milder and therefore have poorer recall. Similarly, exposure misclassification from self‐reported antibiotic use may have occurred, with the proportion of mothers reporting antibiotic use during pregnancy lower than registry‐based estimates for the Norwegian population [32]. These differences may also reflect the measurement of antibiotic use rather than prescription dispensation. Finally, the information did not allow investigation of specific infectious pathogens, nor could we explore or account for hospital proximity, as residency data were not available.

Regarding the population analysed, Norway is a high‐income country with relatively low inequality [33], a relatively low proportion of caesarean births [34], and high rates of breastfeeding initiation [35]. The proportion of births by caesarean in our cohort was comparable with the Norwegian population [36], while breastfeeding for at least 6 months was slightly higher than other estimates for Norway (~77%–80%) [37]. Additionally, MoBa participants differed from non‐participants on demographic and health characteristics, including on average higher education and maternal age than the Norwegian population [38, 39]. These differences may impact the external validity of the findings, particularly in settings with different exposure prevalences. We did not consider the role of daycare attendance in this study, as most Norwegian children do not attend in the first year of life [40]. Overall, replication in large populations with different characteristics, particularly in relation to the exposures, is warranted. The loss to follow‐up in MoBa showed patterns across important maternal factors and for breastfeeding duration. However, we observed only small differences between unweighted and weighted analyses accounting for this potential selection bias (Table S1).

### 4.4 Interpretation

Our findings of an increased risk of hospitalisation for infection following prenatal antibiotic exposure and caesarean birth align with previous registry‐based analyses. Studies in Sweden, Denmark, and France have found increased risks, ranging between 12% to 28%, of severe infections (hospitalised or outpatient specialist care) in children exposed to prenatal antibiotics [4, 5, 6]. The Swedish study also showed a 34% increased rate of antibiotic prescriptions (a marker of non‐hospitalised bacterial infections) among exposed children in the first year of life [5]. We did not include antibiotic prescriptions as an outcome as we were interested in overall infections, many of which are viral. A systematic review and meta‐analysis of caesarean birth and childhood infection outcomes found caesarean birth was associated with a 9%–20% increased risk of hospitalised infections overall, and for respiratory and gastrointestinal infection sub‐groups [7].

Given the higher risk of hospitalisation for infection reported for prenatal antibiotic exposure and caesarean birth, our finding of a very small increased rate of infection episodes associated with these exposures was a surprising and reassuring finding. Two previous cohort studies examined the association between prenatal antibiotic exposure and less severe infection outcomes. One reported a higher rate of otitis media, and the other higher tonsillitis, pneumonia, helicobacter, and urinary tract infections, but not otitis media or gastroenteritis [13, 14]. Both studies were in much smaller cohorts and consequently had wide confidence intervals. The systematic review of caesarean births and infections summarised studies on less severe infections [7], including three small cohort studies [15, 16, 18] on general infection episodes, which showed results on either side of the null and modest effects.

Breastfeeding protects against infections [8, 9, 10, 11], with evidence including data from the MoBa cohort [12]. This previous MoBa study analysed the association of breastfeeding duration with frequent infections (> 10 episodes) and with hospitalisation for infection from birth to 18 months of age. They found higher risks of both outcomes for breastfeeding durations of 6 months or less compared to 12 or more months [12]. Our study provides novel analysis on the effects of shorter breastfeeding duration according to prior adverse exposures in the perinatal period. Although we did not observe evidence of effect modification, the pattern of increasing risk seen with cumulative adverse exposures suggests that breastfeeding promotion could be particularly important (where feasible) in children with high infection risk following perinatal exposures. If these associations represent causal effects, this would indicate that maintained breastfeeding (where possible or chosen) may counteract some of the risk for infants exposed to adverse perinatal factors and warrants further exploration. The proportion of children breastfed for at least 6 months is high in this cohort and in Norway generally. Policies to support breastfeeding remain important, particularly in other settings with lower breastfeeding rates.

## 5 Conclusions

In summary, we observed only small differences in the number of infections from birth to 36 months age associated with prenatal antibiotic exposure, caesarean birth, and shorter breastfeeding duration. We found an increased risk of hospitalisation for infection in children associated with each exposure, in alignment with previous studies. Additionally, we showed a cumulative increased risk of hospitalisation for infection in children with multiple adverse exposures. Our findings contribute to our understanding of early life exposures that may influence differential infection severity in children.

## Author Contributions

All authors contributed to the study conception and design. I.M.F.T. performed the data analysis and wrote the first draft of the manuscript. All authors contributed to interpreting the analyses, critically revising the manuscript and approving of the final draft.

## Funding

I.M.F.T. is supported by an Australian Government Research Training Program (RTP) Scholarship. M.C.M. is supported by the Research Council of Norway through its Centres of Excellence funding scheme (project number 262700). D.P.B. is supported by a NHMRC Investigator Grant (GTN1175744). Research at the Murdoch Children's Research Institute is supported by the Victorian Government's Operational Infrastructure Support Program.

## Consent

All MoBa participants provided written informed consent to participate in the study, including linkage with health registry data. Participants were free to withdraw from the study and/or have all their data deleted at any time. This study only used data for participants with ongoing consent.

## Conflicts of Interest

The authors declare no conflicts of interest.

## Supporting information


**Table S1:** Comparison of unweighted and weighted analyses.
**Table S2:** Descriptive statistics for 6‐month timepoint study population.
**Table S3:** Descriptive statistics for 18‐month timepoint study population.
**Table S4:** Comparison of characteristics for those lost to follow up with those remaining in the study between 6‐month and 36‐month timepoints.
**Table S5:** Association of breastfeeding for less than 6 months compared to 6 or more months with the number of infections and hospital admission for infections across prenatal antibiotic exposure and mode of birth strata.
**Figure S1:** Results for respiratory infections only.
**Figure S2:** Results for number of infections excluding common colds.
**Figure S3:** Results for 0–6‐months, 6–18 months and 18–36 months age windows separately for children with complete follow up to 36 months age.
**Figure S4:** Results for exclusive breastfeeding as exposure variable.
**Figure S5:** Results for emergency and elective caesarean births separately.

## Data Availability

Data from the Norwegian Mother, Father and Child Cohort Study and the Medical Birth Registry of Norway used in this study are managed by the national health register holders in Norway (Norwegian Institute of Public Health) and can be made available to researchers, provided approval from the Regional Committees for Medical and Health Research Ethics (REC), compliance with the EU General Data Protection Regulation (GDPR) and approval from the data owners. The consent given by the participants does not allow for the storage of data on an individual level in repositories or journals. Researchers who want access to data sets for replication should apply through helsedata.no. Access to datasets requires approval from the Regional Committee for Medical and Health Research Ethics in Norway and an agreement with MoBa.
