# Supplementary material for: Joint Effects of Prenatal Antibiotics, Mode of Birth and Breastfeeding Duration on Childhood Infections: The Norwegian MoBa Cohort Study
Source: Paediatr Perinat Epidemiol. 2026 Feb 23;40(5):608–19. doi: 10.1111/ppe.70120 (PMC13423271; doi:10.1111/ppe.70120)
Supplement: Supplementary file 1 — Table S1: Comparison of unweighted and weighted analyses. Table S2: Descriptive statistics for 6‐month timepoint study population. Table S3: Descriptive statistics for 18‐month timepoint study population. Table S4: Comparison of characteristics for those lost to follow up with those remaining in the study between 6‐month and 36‐month timepoints. Table S5: Association of breastfeeding for less than 6 months compared to 6 or more months with the number of infections and hospital admission for infections across prenatal antibiotic exposure and mode of birth strata. Figure S1: Results for respiratory infections only. Figure S2: Results for number of infections excluding common colds. Figure S3: Results for 0–6‐months, 6–18 months and 18–36 months age windows separately for children with complete follow up to 36 months age. Figure S4: Results for exclusive breastfeeding as exposure variable. Figure S5: Results for emergency and elective caesarean births separately. [file PPE-40-608-s001.docx]

# Supplementary material

**Contents:**

- **Supplementary Table 1** – Comparison of unweighted and weighted analyses
- **Supplementary Table 2** – Descriptive statistics for 6-month timepoint study population.
- **Supplementary Table 3** – Descriptive statistics for 18-month timepoint study population.
- **Supplementary Table 4 -** Comparison of characteristics for those lost to follow up with those remaining in the study between 6-month and 36-month timepoints.
- **Supplementary Table 5** – Association of breastfeeding for less than 6 months compared to 6 or more months with the number of infections and hospital admission for infections across prenatal antibiotic exposure and mode of birth strata.
- **Supplementary Figure 1 –** Results for respiratory infections only.
- **Supplementary Figure 2** – Results for number of infections excluding common colds
- **Supplementary Figure 3** – Results for 0-6-months, 6-18 months and 18-36 months age windows separately for children with complete follow up to 36 months age.
- **Supplementary Figure 4 –** Results for exclusive breastfeeding as exposure variable
- **Supplementary Figure 5** – Results for emergency and elective caesarean births separately

**Supplementary Table 1 –** Comparison of unweighted (complete case) and inverse probability weighted analyses to account for loss to follow up for main analysis from birth to 36 months of age. BF = breastfeeding.

| **Exposure** | **Number of infections unweighted analysis**  **IRR (95% CI)** | **Number of infections**  **weighted analysis**  **IRR (95% CI)** | **Hospitalisation**  **for infection unweighted analysis**  **RR (95% CI)** | **Hospitalisation**  **for infection weighted analysis**  **RR (95% CI)** |
| --- | --- | --- | --- | --- |
| **Prenatal antibiotic exposure** | | | | |
| No prenatal antibiotics | 1.00 (Reference) | 1.00 (Reference) | 1.00 (Reference) | 1.00 (Reference) |
| Prenatal antibiotics | 1.04 (1.02-1.05) | 1.04 (1.02-1.05) | 1.11 (1.05-1.18) | 1.11 (1.05-1.17) |
| **Mode of birth** | | | | |
| Vaginal | 1.00 (Reference) | 1.00 (Reference) | 1.00 (Reference) | 1.00 (Reference) |
| Caesarean | 1.01 (1.00-1.03) | 1.02 (1.00-1.03) | 1.19 (1.13-1.26) | 1.20 (1.14-1.27) |
| **Breastfeeding duration** | | | | |
| Breastfeeding ≥6 months | 1.00 (Reference) | 1.00 (Reference) | 1.00 (Reference) | 1.00 (Reference) |
| Breastfeeding <6 months | 1.04 (1.02-1.05) | 1.04 (1.02-1.05) | 1.16 (1.10-1.21) | 1.16 (1.10-1.22) |
| **Combinations of prenatal antibiotic exposure and mode of birth** | | | | |
| No prenatal antibiotics, vaginal | 1.00 (Reference) | 1.00 (Reference) | 1.00 (Reference) | 1.00 (Reference) |
| Prenatal antibiotics, vaginal | 1.03 (1.02-1.05) | 1.03 (1.01-1.05) | 1.11 (1.05-1.18) | 1.10 (1.04-1.17) |
| No prenatal antibiotics, caesarean | 1.01 (0.99-1.03) | 1.01 (1.00-1.03) | 1.19 (1.13-1.26) | 1.19 (1.13-1.26) |
| Prenatal antibiotics, caesarean | 1.07 (1.03-1.12) | 1.07 (1.03-1.11) | 1.34 (1.19-1.51) | 1.36 (1.20-1.54) |
| **Combinations of all three exposures** | | | | |
| No prenatal antibiotics, vaginal, BF≥6m | 1.00 (Reference) | 1.00 (Reference) | 1.00 (Reference) | 1.00 (Reference) |
| No prenatal antibiotics, vaginal, BF<6m | 1.04 (1.02-1.06) | 1.04 (1.02-1.06) | 1.16 (1.10-1.24) | 1.17 (1.10-1.25) |
| Prenatal antibiotics, vaginal, BF≥6m | 1.06 (1.04-1.08) | 1.06 (1.04-1.08) | 1.14 (1.07-1.22) | 1.14 (1.07-1.22) |
| Prenatal antibiotics, vaginal, BF<6m | 1.06 (1.02-1.11) | 1.07 (1.02-1.11) | 1.18 (1.03-1.36) | 1.19 (1.04-1.37) |
| No prenatal antibiotics, caesarean, BF≥6m | 1.00 (0.99-1.02) | 1.01 (0.99-1.03) | 1.13 (1.06-1.20) | 1.12 (1.05-1.20) |
| No prenatal antibiotics, caesarean, BF<6m | 1.05 (1.01-1.08) | 1.05 (1.01-1.08) | 1.34 (1.21-1.48) | 1.31 (1.18-1.45) |
| Prenatal antibiotics, caesarean, BF≥6m | 1.10 (1.06-1.15) | 1.10 (1.06-1.15) | 1.30 (1.12-1.50) | 1.30 (1.12-1.51) |
| Prenatal antibiotics, caesarean, BF<6m | 1.10 (1.01-1.20) | 1.09 (1.00-1.19) | 1.53 (1.23-1.90) | 1.55 (1.24-1.93) |

**Supplementary Table 2.** Descriptive statistics by exposures for study population analysed at 6-month timepoint

|  | **Prenatal antibiotics** | | **Mode of birth** | | **Breastfeeding** | |
| --- | --- | --- | --- | --- | --- | --- |
|  | **No** | **Yes** | **Vaginal** | **Caesarean** | **≥6 months** | **<6 months** |
|  | 62737 (87.7%) | 8794 (12.3%) | 61799 (86.4%) | 9732 (13.6%) | 59702 (83.5%) | 11829 (16.5%) |
| **Maternal and pregnancy factors** | | | | | | |
| Maternal age |  |  |  |  |  |  |
| *<20* | 432 (0.7) | 64 (0.7) | 442 (0.7) | 54 (0.6) | 259 (0.4) | 237 (2.0) |
| *20-24* | 5815 (9.3) | 853 (9.7) | 5920 (9.6) | 748 (7.7) | 4626 (7.7) | 2042 (17.3) |
| *25-29* | 20997 (33.5) | 2836 (32.2) | 20971 (33.9) | 2862 (29.4) | 19713 (33.0) | 4120 (34.8) |
| *30-34* | 24517 (39.1) | 3521 (40.0) | 24236 (39.2) | 3802 (39.1) | 24336 (40.8) | 3702 (31.3) |
| *35+* | 10976 (17.5) | 1520 (17.3) | 10230 (16.6) | 2266 (23.3) | 10768 (18.0) | 1728 (14.6) |
| Maternal education |  |  |  |  |  |  |
| *1–2-year high school* | 2549 (4.1) | 325 (3.7) | 2417 (3.9) | 457 (4.7) | 1859 (3.1) | 1015 (8.6) |
| *3-year high school* | 7296 (11.6) | 913 (10.4) | 6935 (11.2) | 1274 (13.1) | 6217 (10.4) | 1992 (16.8) |
| *9-year high school* | 1022 (1.6) | 129 (1.5) | 965 ( 1.6) | 186 (1.9) | 696 (1.2) | 455 (3.8) |
| *Vocational high school* | 7659 (12.2) | 1094 (12.4) | 7410 (12.0) | 1343 (13.8) | 6156 (10.3) | 2597 (22.0) |
| *Bachelor’s degree* | 27196 (43.3) | 4012 (45.6) | 27210 (44.0) | 3998 (41.1) | 27002 (45.2) | 4206 (35.6) |
| *Master’s degree or higher* | 17015 (27.1) | 2321 (26.4) | 16862 (27.3) | 2474 (25.4) | 17772 (29.8) | 1564 (13.2) |
| Parity = nulliparous, n (%) | 29495 (47.0) | 3714 (42.2) | 28048 (45.4) | 5161 (53.0) | 26996 (45.2) | 6213 (52.5) |
| BMI before pregnancy |  |  |  |  |  |  |
| *<18.5* | 1847 (2.9) | 249 (2.8) | 1871 (3.0) | 225 (2.3) | 1712 (2.9) | 384 (3.2) |
| *18.5-24.9* | 41470 (66.1) | 5624 (64.0) | 41675 (67.4) | 5419 (55.7) | 40864 (68.4) | 6230 (52.7) |
| *25-29.9* | 13653 (21.8) | 1942 (22.1) | 13058 (21.1) | 2537 (26.1) | 12486 (20.9) | 3109 (26.3) |
| *30+* | 5767 (9.2) | 979 (11.1) | 5195 (8.4) | 1551 (15.9) | 4640 (7.8) | 2106 (17.8) |
| Smoking |  |  |  |  |  |  |
| *Never smoker* | 32386 (51.6) | 4652 (52.9) | 32334 (52.3) | 4704 (48.3) | 32424 (54.3) | 4614 (39.0) |
| *Former smoker* | 13687 (21.8) | 1830 (20.8) | 13392 (21.7) | 2125 (21.8) | 13300 (22.3) | 2217 (18.7) |
| *Before pregnancy* | 11847 (18.9) | 1612 (18.3) | 11433 (18.5) | 2026 (20.8) | 10607 (17.8) | 2852 (24.1) |
| *Early pregnancy* | 920 (1.5) | 126 (1.4) | 847 (1.4) | 199 (2.0) | 718 (1.2) | 328 (2.8) |
| *During pregnancy* | 3897 (6.2) | 574 (6.5) | 3793 (6.1) | 678 (7.0) | 2653 (4.4) | 1818 (15.4) |
| Diabetes |  |  |  |  |  |  |
| *No diabetes* | 61841 (98.6) | 8621 (98.0) | 61047 (98.8) | 9415 (96.7) | 58909 (98.7) | 11553 (97.7) |
| *Pre-gestational diabetes* | 379 (0.6) | 69 ( 0.8) | 293 (0.5) | 155 (1.6) | 328 (0.5) | 120 (1.0) |
| *Gestational diabetes* | 517 (0.8) | 104 (1.2) | 459 (0.7) | 162 (1.7) | 465 (0.8) | 156 (1.3) |
| Hypertension, n (%) | 1531 (2.4) | 247 (2.8) | 1411 (2.3) | 367 (3.8) | 1404 (2.4) | 374 (3.2) |
| Pre-eclampsia /eclampsia/HELLP, n (%) | 2282 (3.6) | 328 (3.7) | 1730 (2.8) | 880 (9.0) | 1974 (3.3) | 636 (5.4) |
| Infection during pregnancy = yes, n (%) | 47868 (76.3) | 8545 (97.2) | 48782 (78.9) | 7631 (78.4) | 47213 (79.1) | 9200 (77.8) |
| Year of birth |  |  |  |  |  |  |
| *2002* | 3796 (6.1) | 531 (6.0) | 3775 (6.1) | 552 (5.7) | 3566 (6.0) | 761 (6.4) |
| *2003* | 8238 (13.1) | 1241 (14.1) | 8291 (13.4) | 1188 (12.2) | 7796 (13.1) | 1683 (14.2) |
| *2004* | 8870 (14.1) | 1234 (14.0) | 8737 (14.1) | 1367 (14.0) | 8346 (14.0) | 1758 (14.9) |
| *2005* | 10004 (15.9) | 1449 (16.5) | 9849 (15.9) | 1604 (16.5) | 9515 (15.9) | 1938 (16.4) |
| *2006* | 11121 (17.7) | 1463 (16.6) | 10801 (17.5) | 1783 (18.3) | 10573 (17.7) | 2011 (17.0) |
| *2007* | 10060 (16.0) | 1430 (16.3) | 9881 (16.0) | 1609 (16.5) | 9624 (16.1) | 1866 (15.8) |
| *2008* | 8508 (13.6) | 1155 (13.1) | 8349 (13.5) | 1314 (13.5) | 8173 (13.7) | 1490 (12.6) |
| *2009* | 2140 (3.4) | 291 (3.3) | 2116 (3.4) | 315 (3.2) | 2109 (3.5) | 322 (2.7) |
| **Child factors** | | | | | | |
| Child sex = male, n (%) | 32011 (51.0) | 4510 (51.3) | 31289 (50.6) | 5232 (53.8) | 30265 (50.7) | 6256 (52.9) |
| Gestational age (weeks) |  |  |  |  |  |  |
| *<32* | 207 (0.3) | 19 (0.2) | 71 (0.1) | 155 (1.6) | 149 (0.2) | 77 (0.7) |
| *32-33* | 369 (0.6) | 57 (0.6) | 214 (0.3) | 212 (2.2) | 311 (0.5) | 115 (1.0) |
| *34-35* | 955 (1.5) | 140 (1.6) | 746 (1.2) | 349 (3.6) | 810 (1.4) | 285 (2.4) |
| *36-37* | 3944 (6.3) | 549 (6.2) | 3517 (5.7) | 976 (10.0) | 3604 (6.0) | 889 (7.5) |
| *38-40* | 39521 (63.0) | 5631 (64.0) | 39467 (63.9) | 5685 (58.4) | 37797 (63.3) | 7355 (62.2) |
| *>40* | 17741 (28.3) | 2398 (27.3) | 17784 (28.8) | 2355 (24.2) | 17031 (28.5) | 3108 (26.3) |
| Birth weight |  |  |  |  |  |  |
| *<2500* | 1522 (2.4) | 211 (2.4) | 984 (1.6) | 749 (7.7) | 1270 (2.1) | 463 (3.9) |
| *2500-2999* | 5363 (8.5) | 755 (8.6) | 5097 (8.2) | 1021 (10.5) | 4904 (8.2) | 1214 (10.3) |
| *3000-3499* | 18397 (29.3) | 2551 (29.0) | 18478 (29.9) | 2470 (25.4) | 17450 (29.2) | 3498 (29.6) |
| *3500-4000* | 23996 (38.2) | 3391 (38.6) | 24192 (39.1) | 3195 (32.8) | 23140 (38.8) | 4247 (35.9) |
| *>4000* | 13459 (21.5) | 1886 (21.4) | 13048 (21.1) | 2297 (23.6) | 12938 (21.7) | 2407 (20.3) |
| **Exposures of interest** | | | | | | |
| Prenatal antibiotics = yes | NA | NA | 7541 (12.2) | 1253 (12.9) | 7232 (12.1) | 1562 (13.2) |
| Caesarean birth | 8479 (13.5) | 1253 (14.2) | NA | NA | 7454 (12.5) | 2278 (19.3) |
| Breastfeeding <6 months | 10267 (16.4) | 1562 (17.8) | 9551 (15.5) | 2278 (23.4) | NA | NA |

**Supplementary Table 3.** Descriptive statistics by exposures for study population analysed at 18-month timepoint

|  | **Prenatal antibiotics** | | **Mode of birth** | | **Breastfeeding** | |
| --- | --- | --- | --- | --- | --- | --- |
|  | **No** | **Yes** | **Vaginal** | **Caesarean** | **≥6 months** | **<6 months** |
|  | 52186 (87.8%) | 7262 (12.2%) | 51400 (86.5%) | 8048 (13.5%) | 50158 (84.4%) | 9290 (15.6%) |
| **Maternal and pregnancy factors** | | | | | | |
| Maternal age |  |  |  |  |  |  |
| *<20* | 282 (0.5) | 45 (0.6) | 286 (0.6) | 41 (0.5) | 176 (0.4) | 151 (1.6) |
| *20-24* | 4532 (8.7) | 648 (8.9) | 4600 (8.9) | 580 (7.2) | 3704 (7.4) | 1476 (15.9) |
| *25-29* | 17426 (33.4) | 2378 (32.7) | 17476 (34.0) | 2328 (28.9) | 16576 (33.0) | 3228 (34.7) |
| *30-34* | 20690 (39.6) | 2940 (40.5) | 20446 (39.8) | 3184 (39.6) | 20608 (41.1) | 3022 (32.5) |
| *35+* | 9256 (17.7) | 1251 (17.2) | 8592 (16.7) | 1915 (23.8) | 9094 (18.1) | 1413 (15.2) |
| Maternal education |  |  |  |  |  |  |
| *1–2-year high school* | 1947 (3.7) | 249 (3.4) | 1844 (3.6) | 352 (4.4) | 1464 (2.9) | 732 (7.9) |
| *3-year high school* | 5867 (11.2) | 745 (10.3) | 5584 (10.9) | 1028 (12.8) | 5099 (10.2) | 1513 (16.3) |
| *9-year high school* | 723 (1.4) | 91 (1.3) | 679 (1.3) | 135 (1.7) | 511 (1.0) | 303 (3.3) |
| *Vocational high school* | 6121 (11.7) | 852 (11.7) | 5879 (11.4) | 1094 (13.6) | 4991 (10.0) | 1982 (21.3) |
| *Bachelor’s degree* | 23003 (44.1) | 3339 (46.0) | 23011 (44.8) | 3331 (41.4) | 22866 (45.6) | 3476 (37.4) |
| *Master’s degree or higher* | 14525 (27.8) | 1986 (27.3) | 14403 (28.0) | 2108 (26.2) | 15227 (30.4) | 1284 (13.8) |
| Parity = nulliparous, n (%) | 25081 (48.1) | 3131 (43.1) | 23813 (46.3) | 4399 (54.7) | 23234 (46.3) | 4978 (53.6) |
| BMI before pregnancy |  |  |  |  |  |  |
| *<18.5* | 1537 (2.9) | 209 (2.9) | 1559 (3.0) | 187 (2.3) | 1444 (2.9) | 302 (3.3) |
| *18.5-24.9* | 34699 (66.5) | 4697 (64.7) | 34882 (67.9) | 4514 (56.1) | 34503 (68.8) | 4893 (52.7) |
| *25-29.9* | 11262 (21.6) | 1581 (21.8) | 10763 (20.9) | 2080 (25.8) | 10395 (20.7) | 2448 (26.4) |
| *30+* | 4688 (9.0) | 775 (10.7) | 4196 (8.2) | 1267 (15.7) | 3816 (7.6) | 1647 (17.7) |
| Smoking |  |  |  |  |  |  |
| *Never smoker* | 27532 (52.8) | 3925 (54.0) | 27501 (53.5) | 3956 (49.2) | 27692 (55.2) | 3765 (40.5) |
| *Former smoker* | 11344 (21.7) | 1498 (20.6) | 11079 (21.6) | 1763 (21.9) | 11062 (22.1) | 1780 (19.2) |
| *Before pregnancy* | 9591 (18.4) | 1318 (18.1) | 9267 (18.0) | 1642 (20.4) | 8722 (17.4) | 2187 (23.5) |
| *Early pregnancy* | 727 (1.4) | 94 (1.3) | 664 (1.3) | 157 (2.0) | 582 (1.2) | 239 (2.6) |
| *During pregnancy* | 2992 (5.7) | 427 (5.9) | 2889 (5.6) | 530 (6.6) | 2100 (4.2) | 1319 (14.2) |
| Diabetes |  |  |  |  |  |  |
| *No diabetes* | 51445 (98.6) | 7124 (98.1) | 50780 (98.8) | 7789 (96.8) | 49488 (98.7) | 9081 (97.8) |
| *Pre-gestational diabetes* | 317 (0.6) | 55 ( 0.8) | 240 (0.5) | 132 (1.6) | 279 (0.6) | 93 (1.0) |
| *Gestational diabetes* | 424 (0.8) | 83 (1.1) | 380 (0.7) | 127 (1.6) | 391 (0.8) | 116 (1.2) |
| Hypertension, n (%) | 1282 (2.5) | 212 (2.9) | 1181 (2.3) | 313 (3.9) | 1195 (2.4) | 299 (3.2) |
| Pre-eclampsia /eclampsia/HELLP, n (%) | 1911 (3.7) | 276 (3.8) | 1452 (2.8) | 735 (9.1) | 1676 (3.3) | 511 (5.5) |
| Infection during pregnancy = yes, n (%) | 39838 (76.3) | 7055 (97.1) | 40595 (79.0) | 6298 (78.3) | 39677 (79.1) | 7216 (77.7) |
| Year of birth |  |  |  |  |  |  |
| *2002* | 3276 (6.3) | 473 (6.5) | 3270 (6.4) | 479 (6.0) | 3109 (6.2) | 640 (6.9) |
| *2003* | 6980 (13.4) | 1060 (14.6) | 7031 (13.7) | 1009 (12.5) | 6677 (13.3) | 1363 (14.7) |
| *2004* | 7524 (14.4) | 1038 (14.3) | 7404 (14.4) | 1158 (14.4) | 7126 (14.2) | 1436 (15.5) |
| *2005* | 8301 (15.9) | 1171 (16.1) | 8176 (15.9) | 1296 (16.1) | 7986 (15.9) | 1486 (16.0) |
| *2006* | 9089 (17.4) | 1189 (16.4) | 8825 (17.2) | 1453 (18.1) | 8742 (17.4) | 1536 (16.5) |
| *2007* | 8310 (15.9) | 1141 (15.7) | 8124 (15.8) | 1327 (16.5) | 8005 (16.0) | 1446 (15.6) |
| *2008* | 6974 (13.4) | 960 (13.2) | 6865 (13.4) | 1069 (13.3) | 6795 (13.5) | 1139 (12.3) |
| *2009* | 1732 (3.3) | 230 (3.2) | 1705 (3.3) | 257 (3.2) | 1718 (3.4) | 244 (2.6) |
| **Child factors** | | | | | | |
| Child sex = male, n (%) | 26613 (51.0) | 3688 (50.8) | 26008 (50.6) | 4293 (53.3) | 25396 (50.6) | 4905 (52.8) |
| Gestational age (weeks) |  |  |  |  |  |  |
| *<32* | 179 (0.3) | 18 (0.2) | 62 (0.1) | 135 (1.7) | 133 (0.3) | 64 (0.7) |
| *32-33* | 314 (0.6) | 48 (0.7) | 189 (0.4) | 173 (2.1) | 265 (0.5) | 97 (1.0) |
| *34-35* | 799 (1.5) | 116 (1.6) | 626 (1.2) | 289 (3.6) | 681 (1.4) | 234 (2.5) |
| *36-37* | 3243 (6.2) | 446 (6.1) | 2892 (5.6) | 797 (9.9) | 3004 (6.0) | 685 (7.4) |
| *38-40* | 32845 (62.9) | 4665 (64.2) | 32842 (63.9) | 4668 (58.0) | 31761 (63.3) | 5749 (61.9) |
| *>40* | 14806 (28.4) | 1969 (27.1) | 14789 (28.8) | 1986 (24.7) | 14314 (28.5) | 2461 (26.5) |
| Birth weight |  |  |  |  |  |  |
| *<2500* | 1280 (2.5) | 181 (2.5) | 833 (1.6) | 628 (7.8) | 1087 (2.2) | 374 (4.0) |
| *2500-2999* | 4443 (8.5) | 610 (8.4) | 4225 (8.2) | 828 (10.3) | 4119 (8.2) | 934 (10.1) |
| *3000-3499* | 15394 (29.5) | 2116 (29.1) | 15461 (30.1) | 2049 (25.5) | 14694 (29.3) | 2816 (30.3) |
| *3500-4000* | 19967 (38.3) | 2786 (38.4) | 20088 (39.1) | 2665 (33.1) | 19470 (38.8) | 3283 (35.3) |
| *>4000* | 11102 (21.3) | 1569 (21.6) | 10793 (21.0) | 1878 (23.3) | 10788 (21.5) | 1883 (20.3) |
| **Exposures of interest** | | | | | | |
| Prenatal antibiotics = yes | NA | NA | 6252 (12.2) | 1010 (12.5) | 6040 (12.0) | 1222 (13.2) |
| Caesarean birth | 7038 (13.5) | 1010 (13.9) | NA | NA | 6234 (12.4) | 1814 (19.5) |
| Breastfeeding <6 months | 8068 (15.5) | 1222 (16.8) | 7476 (14.5) | 1814 (22.5) | NA | NA |

**Supplementary Table 4** – Comparison of characteristics for mother-child pairs who had complete baseline data and were lost to follow-up between 6-36 months child age with those remaining in the study to 36 months child age.

|  | **Lost to follow up between 6-36 months** | | ***Continued*** | | |
| --- | --- | --- | --- | --- | --- |
|  | **Yes**  26046  (36.4%) | **No**  45485  (63.6%) |  | **Yes** | **No** |
| **Maternal and pregnancy factors** | | | Year of birth |  |  |
| Maternal age |  |  | *2002* | 1463 (5.6) | 2864 (6.3) |
| *<20* | 306 (1.2) | 190 (0.4) | *2003* | 3208 (12.3) | 6271 (13.8) |
| *20-24* | 3002 (11.5) | 3666 (8.1) | *2004* | 3541 (13.6) | 6563 (14.4) |
| *25-29* | 8674 (33.3) | 15159 (33.3) | *2005* | 4150 (15.9) | 7303 (16.1) |
| *30-34* | 9737 (37.4) | 18301 (40.2) | *2006* | 4747 (18.2) | 7837 (17.2) |
| *35+* | 4327 (16.6) | 8169 (18.0) | *2007* | 4395 (16.9) | 7095 (15.6) |
| Maternal education |  |  | *2008* | 3667 (14.1) | 5996 (13.2) |
| *1–2-year high school* | 1379 (5.3) | 1495 (3.3) | *2009* | 875 (3.4) | 1556 (3.4) |
| *3-year high school* | 3347 (12.9) | 4862 (10.7) | **Child factors** | | |
| *9-year high school* | 620 (2.4) | 531 (1.2) | Child sex = male, n (%) | 13364 (51.3) | 23157 (50.9) |
| *Vocational high school* | 3738 (14.4) | 5015 (11.0) | Gestational age (weeks) |  |  |
| *Bachelor’s degree* | 10663 (40.9) | 20545 (45.2) | *<32* | 61 (0.2) | 165 (0.4) |
| *Master’s degree or higher* | 6299 (24.2) | 13037 (28.7) | *32-33* | 146 (0.6) | 280 (0.6) |
| Parity = nulliparous, n (%) | 11067 (42.5) | 22142 (48.7) | *34-35* | 384 (1.5) | 711 (1.6) |
| BMI before pregnancy |  |  | *36-37* | 1653 (6.3) | 2840 (6.2) |
| *<18.5* | 793 (3.0) | 1303 (2.9) | *38-40* | 16667 (64.0) | 28485 (62.6) |
| *18.5-24.9* | 16702 (64.1) | 30392 (66.8) | *>40* | 7135 (27.4) | 13004 (28.6) |
| *25-29.9* | 5856 (22.5) | 9739 (21.4) | Birth weight |  |  |
| *30+* | 2695 (10.3) | 4051 (8.9) | *<2500* | 582 (2.2) | 1151 (2.5) |
| Smoking |  |  | *2500-2999* | 2207 (8.5) | 3911 (8.6) |
| *Never smoker* | 12339 (47.4) | 24699 (54.3) | *3000-3499* | 7653 (29.4) | 13295 (29.2) |
| *Former smoker* | 5817 (22.3) | 9700 (21.3) | *3500-4000* | 9906 (38.0) | 17481 (38.4) |
| *Before pregnancy* | 5372 (20.6) | 8087 (17.8) | *>4000* | 5698 (21.9) | 9647 (21.2) |
| *Early pregnancy* | 473 (1.8) | 573 (1.3) | **Exposures of interest** | | |
| *During pregnancy* | 2045 (7.9) | 2426 (5.3) | Prenatal antibiotics = yes | 3209 (12.3) | 5585 (12.3) |
| Diabetes |  |  | Caesarean birth | 3607 (13.8) | 6125 (13.5) |
| *No diabetes* | 25652 (98.5) | 44810 (98.5) | Breastfeeding <6 months | 5050 (19.4) | 6779 (14.9) |
| *Pre-gestational diabetes* | 159 (0.6) | 289 (0.6) |  |  |  |
| *Gestational diabetes* | 235 (0.9) | 386 (0.8) |  |  |  |
| Hypertension, n (%) | 618 (2.4) | 1160 (2.6) |  |  |  |
| Pre-eclampsia /eclampsia/ HELLP, n (%) | 916 (3.5) | 1694 (3.7) |  |  |  |
| Infection during pregnancy  = yes, n (%) | 20494 (78.7) | 35919 (79.0) |  |  |  |

**Supplementary Table 5** – Association of breastfeeding for less than 6 months compared to 6 or more months with the number of infections and hospital admission for infections across prenatal antibiotic exposure and mode of birth strata

|  | **Breastfeeding (BF)**  **6+ months** | | **Breastfeeding**  **<6 months** | | **Ratio (95% CI) for BF<6m vs BF 6m+ within strata of antibiotics and**  **mode of birth** |
| --- | --- | --- | --- | --- | --- |
|  | N | Effect estimate (95% CI) | N | Effect estimate  (95% CI) |  |
| **Number of infections 0-6m** | | | | | |
| No prenatal antibiotics, vaginal | 45947 | 1.00 (Reference) | 8311 | 1.07 (1.05-1.09) | 1.07 (1.05-1.09) |
| Prenatal antibiotics, vaginal | 6301 | 1.07 (1.05-1.10) | 1240 | 1.13 (1.07-1.19) | 1.06 (1.00-1.12) |
| No prenatal antibiotics, caesarean | 6523 | 0.99 (0.96-1.01) | 1956 | 1.08 (1.03-1.13) | 1.09 (1.03-1.14) |
| Prenatal antibiotics, caesarean | 931 | 1.09 (1.03-1.16) | 322 | 1.11 (1.00-1.24) | 0.99 (0.88-1.13) |
| **Number of infections 0-18m** | | | | | |
| No prenatal antibiotics, vaginal | 38647 | 1.00 (Reference) | 6501 | 1.07 (1.05-1.08) | 1.07 (1.05-1.08) |
| Prenatal antibiotics, vaginal | 5277 | 1.06 (1.04-1.08) | 975 | 1.10 (1.06-1.14) | 1.05 (1.00-1.09) |
| No prenatal antibiotics, caesarean | 5471 | 1.01 (0.99-1.03) | 1567 | 1.07 (1.04-1.10) | 1.06 (1.02-1.09) |
| Prenatal antibiotics, caesarean | 763 | 1.09 (1.04-1.13) | 247 | 1.08 (1.00-1.17) | 0.99 (0.90-1.08) |
| **Number of infections 0-36m** | | | | | |
| No prenatal antibiotics, vaginal | 29802 | 1.00 (Reference) | 4749 | 1.04 (1.02-1.06) | 1.04 (1.02-1.06) |
| Prenatal antibiotics, vaginal | 4102 | 1.06 (1.04-1.08) | 707 | 1.07 (1.02-1.11) | 1.00 (0.96-1.05) |
| No prenatal antibiotics, caesarean | 4204 | 1.01 (0.99-1.03) | 1145 | 1.05 (1.01-1.08) | 1.04 (1.00-1.08) |
| Prenatal antibiotics, caesarean | 598 | 1.10 (1.06-1.15) | 178 | 1.09 (1.00-1.19) | 0.98 (0.89-1.08) |
| **Hospital admission for infection 0-6m** | | | | | |
| No prenatal antibiotics, vaginal | 45947 | 1.00 (Reference) | 8311 | 1.46 (1.30-1.64) | 1.42 (1.26-1.60) |
| Prenatal antibiotics, vaginal | 6301 | 1.35 (1.18-1.53) | 1240 | 1.82 (1.44-2.30) | 1.34 (1.02-1.75) |
| No prenatal antibiotics, caesarean | 6523 | 1.19 (1.03-1.37) | 1956 | 1.56 (1.26-1.94) | 1.47 (1.15-1.88) |
| Prenatal antibiotics, caesarean | 931 | 1.56 (1.14-2.14) | 322 | 1.74 (1.10-2.77) | 1.09 (0.58-2.02) |
| **Hospital admission for infection 0-18m** | | | | | |
| No prenatal antibiotics, vaginal | 38647 | 1.00 (Reference) | 6501 | 1.26 (1.17-1.37) | 1.24 (1.15-1.35) |
| Prenatal antibiotics, vaginal | 5277 | 1.20 (1.10-1.30) | 975 | 1.39 (1.17-1.65) | 1.17 (0.97-1.41) |
| No prenatal antibiotics, caesarean | 5471 | 1.12 (1.02-1.22) | 1567 | 1.55 (1.36-1.77) | 1.49 (1.27-1.75) |
| Prenatal antibiotics, caesarean | 763 | 1.32 (1.07-1.62) | 247 | 1.91 (1.44-2.55) | 1.21 (0.84-1.75) |
| **Hospital admission for infection 0-36m** | | | | | |
| No prenatal antibiotics, vaginal | 29802 | 1.00 (Reference) | 4749 | 1.17 (1.10-1.25) | 1.16 (1.09-1.23) |
| Prenatal antibiotics, vaginal | 4102 | 1.14 (1.07-1.22) | 707 | 1.19 (1.04-1.37) | 1.04 (0.90-1.22) |
| No prenatal antibiotics, caesarean | 4204 | 1.12 (1.05-1.20) | 1145 | 1.31 (1.18-1.45) | 1.22 (1.09-1.38) |
| Prenatal antibiotics, caesarean | 598 | 1.30 (1.12-1.51) | 178 | 1.55 (1.24-1.93) | 1.12 (0.85-1.49) |

Results for respiratory infections only

**Supplementary Figure 1 –** Associations of exposures with number of respiratory infections and hospital admission for respiratory infections from birth to 36 months age. (A) Associations for individual exposures. (B) Associations for combinations of prenatal antibiotic exposure and mode of birth. (C) Association for combinations of all three exposures.

| **(A)**  **(A)** | 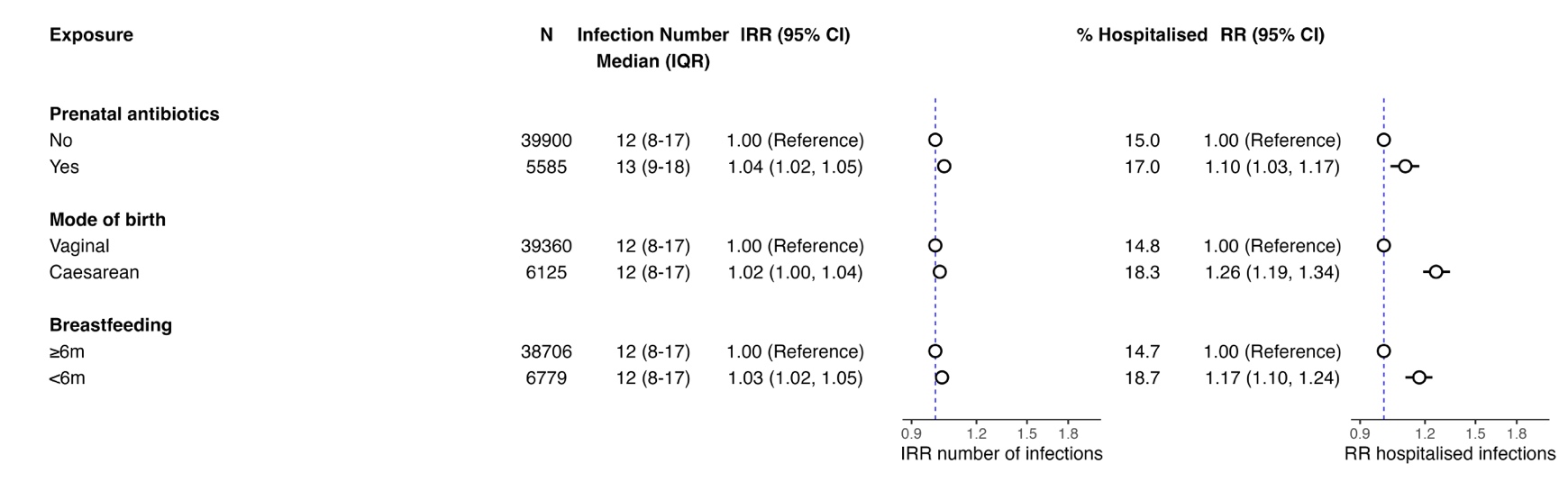 |
| --- | --- |
| **(B)** | 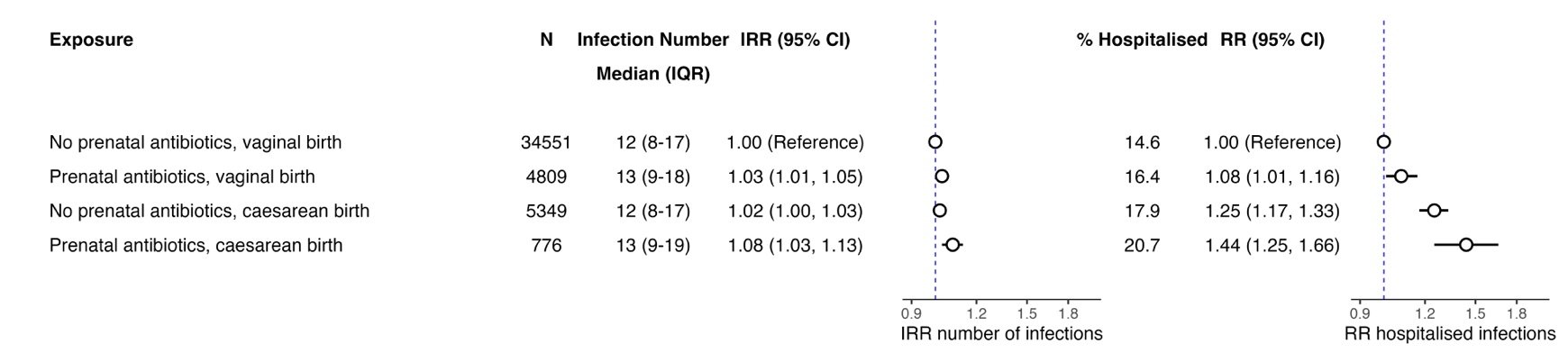 |
| **(C)** | 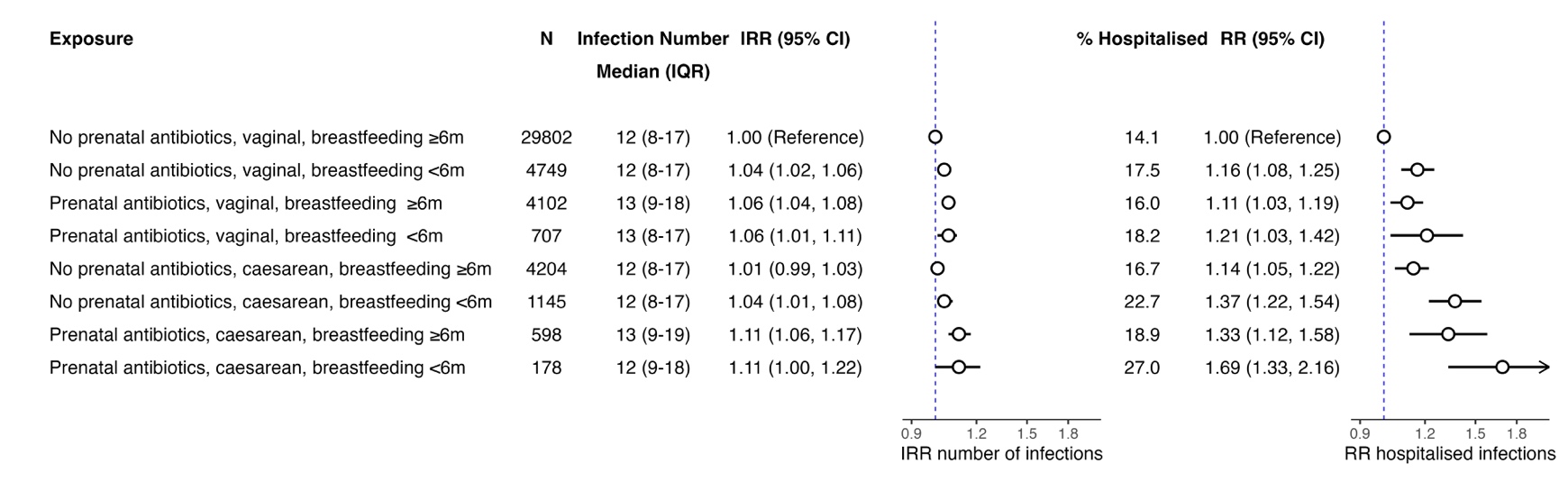 |

Results for number of infections excluding common cold

**Supplementary Figure 2** – Associations of exposures with number of infections excluding common colds from birth to 36 months age. (A) Associations for individual exposures. (B) Associations for combinations of prenatal antibiotic exposure and mode of birth. (C) Association for combinations of all three exposures.

| **(A)**  **(A)** | 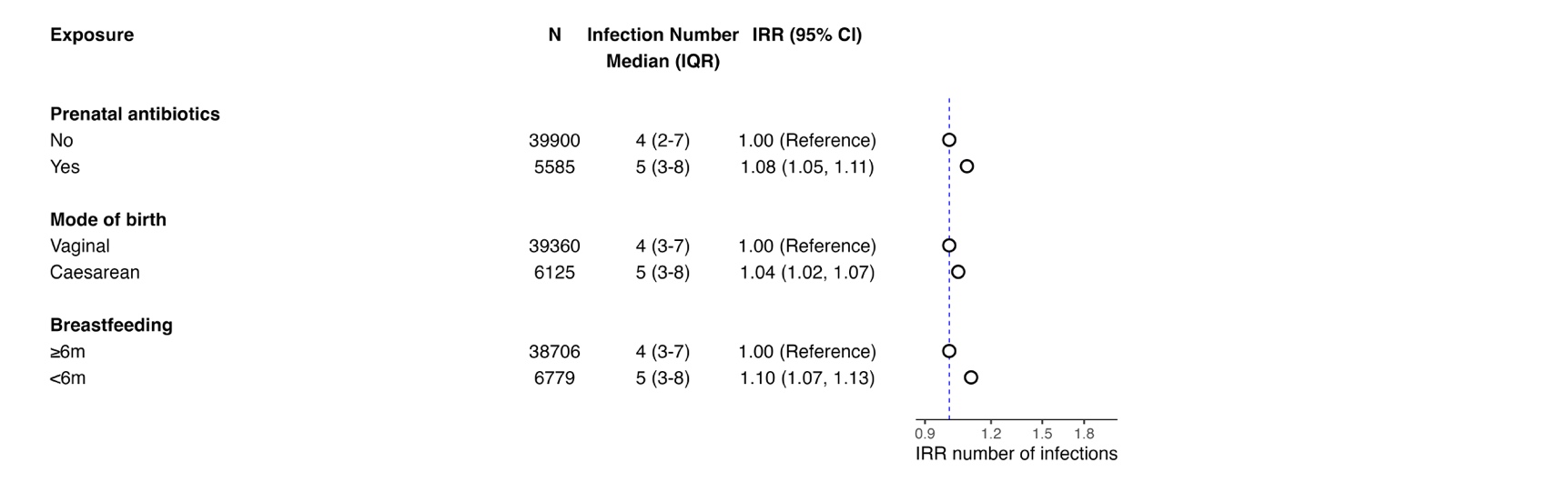 |
| --- | --- |
| **(B)** | 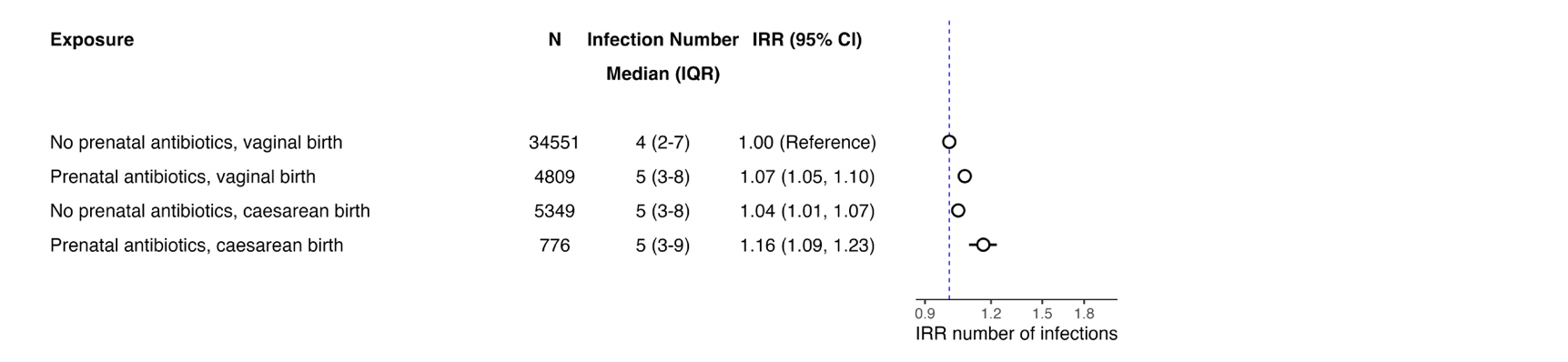 |
| **(C)** | 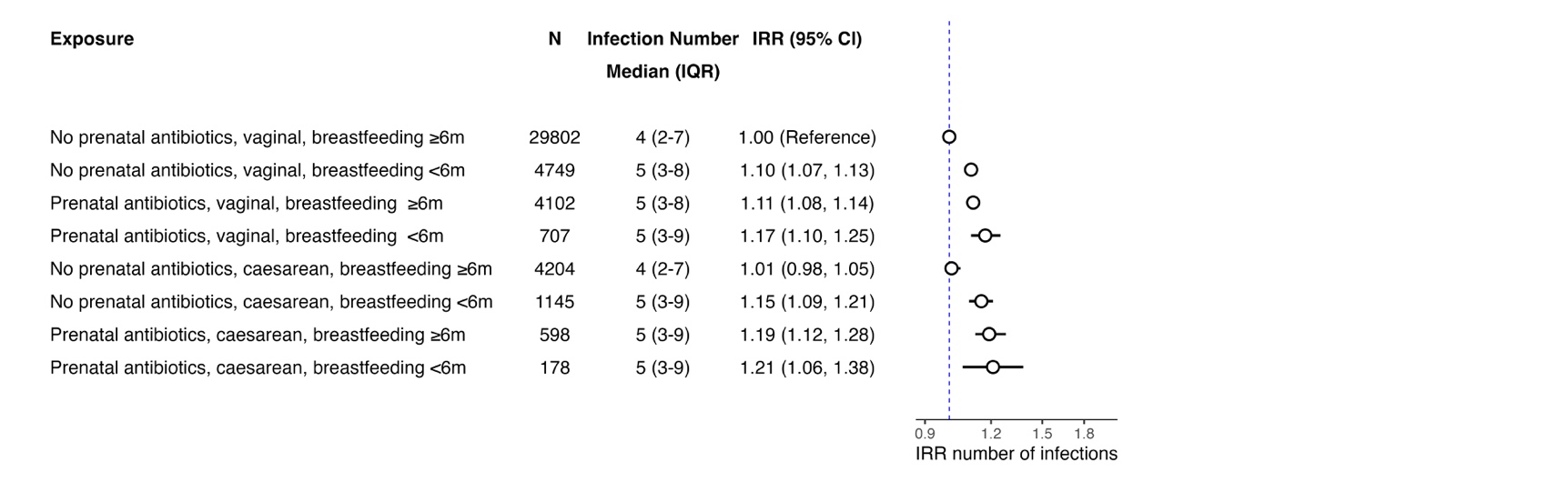 |

Results for 0-6 months, 6-18 months, and 18-36 months age windows separately in children with complete follow-up to 36 months age (n=45,485)

**Supplementary Figure 3 –** Associations of exposures with number of infections and hospital admission for infections from birth to 6 months age, 6 to 18 months age and 18 to 36 months age in children with complete follow-up to 36 months age (n=45,485). (A) Associations for prenatal antibiotic exposure (B) Associations for mode of birth (C) Associations for breastfeeding duration.

| **(A)**  **(A)** | 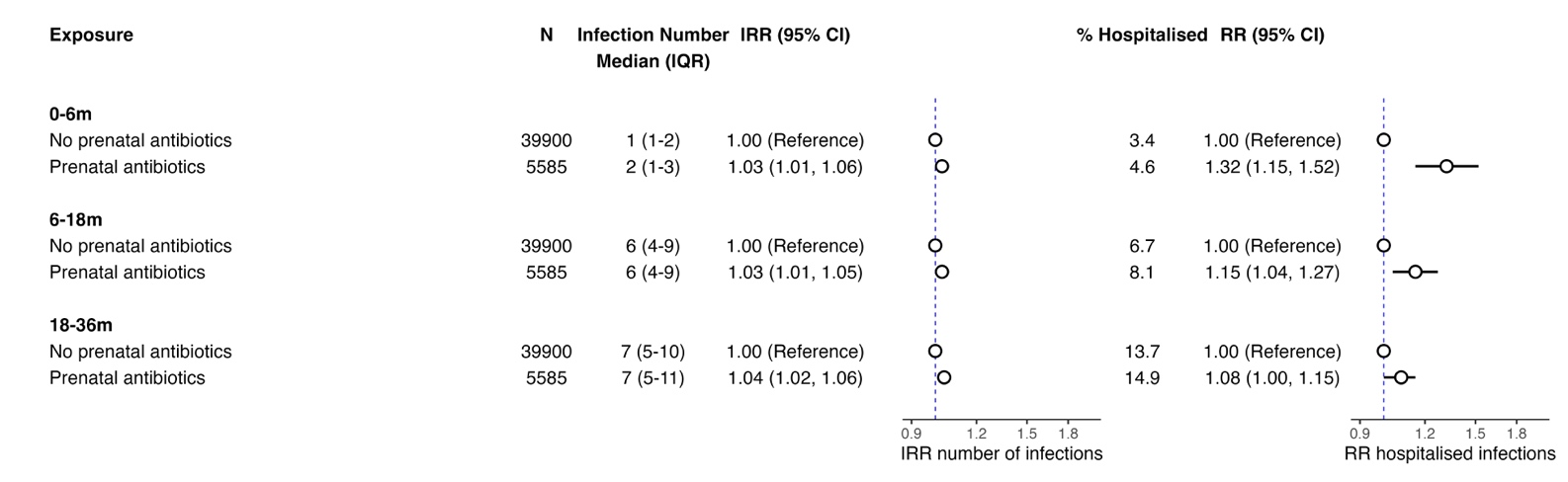 |
| --- | --- |
| **(B)** | 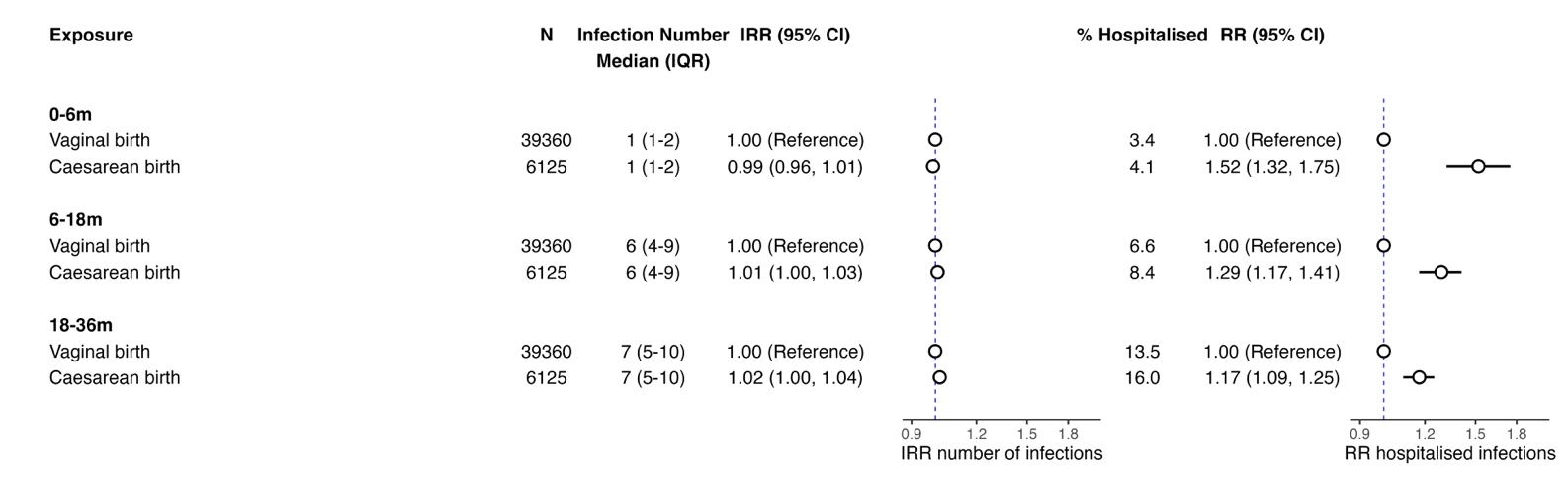 |
| **(C)** | 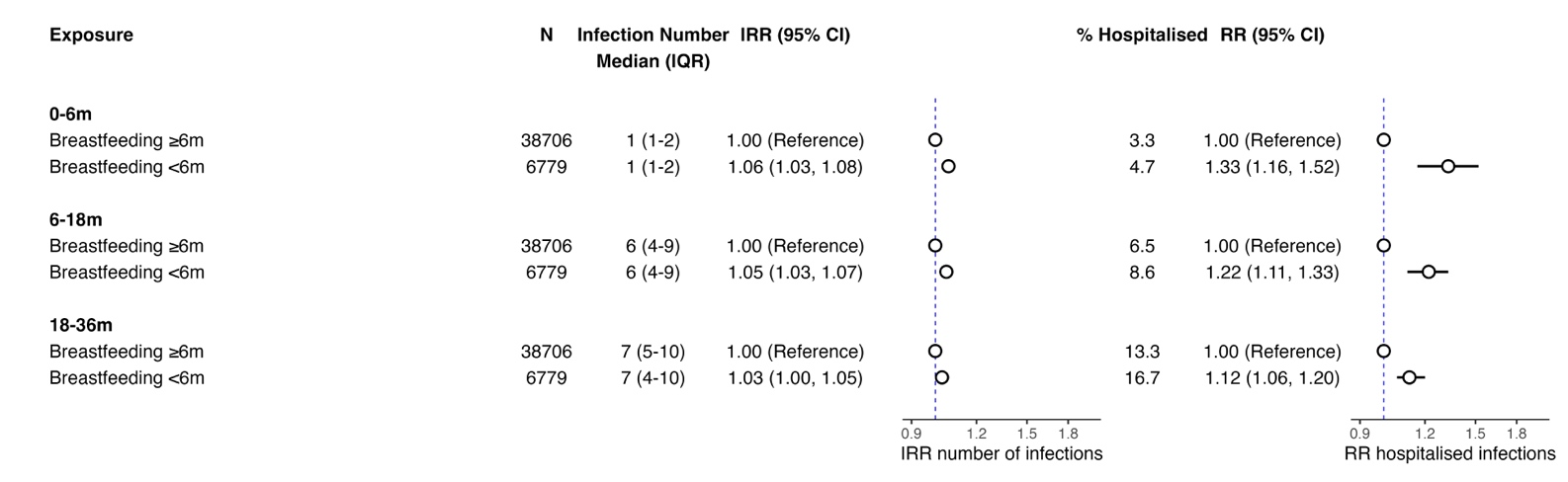 |

Results for exclusive breastfeeding <6 months compared to exclusive breastfeeding 6+ months as breastfeeding exposure variable

**Supplementary Figure 4 –** Associations of exposures with number of infections and hospital admission for infections from birth to 18 months age. (A) Associations for individual exposures. (B) Association for combinations of all three exposures.

| **(A)**  **(A)** | 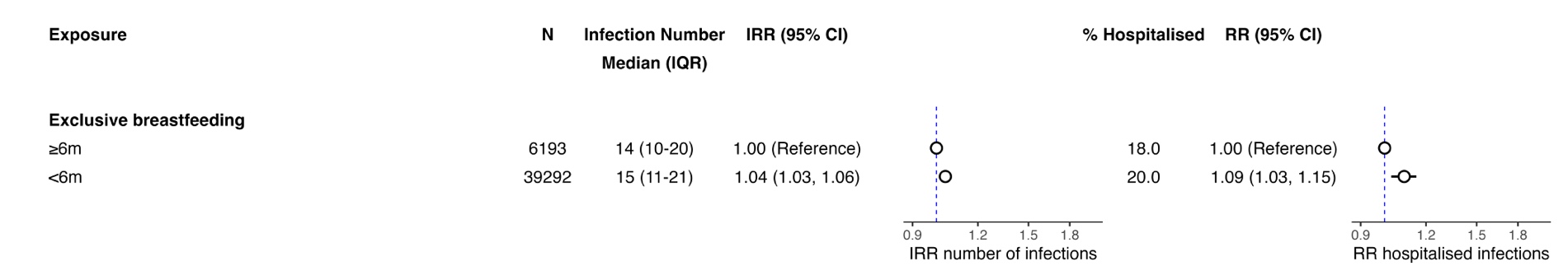 |
| --- | --- |
| **(B)** | 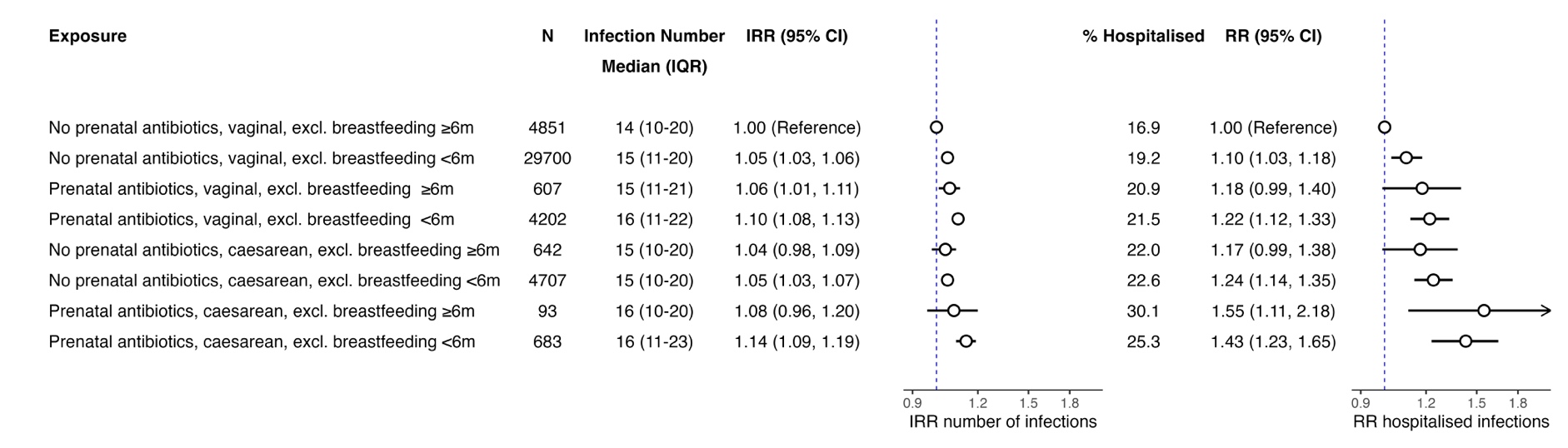 |

Results for emergency and elective caesarean births examined separately

**Supplementary Figure 5 –** Associations of exposures with number of infections and hospital admission for infections from birth to 36 months age. (A) Associations for individual exposures. (B) Associations for combinations of prenatal antibiotic exposure and mode of birth. (C) Association for combinations of all three exposures. CS = caesarean section

| **(A)** | 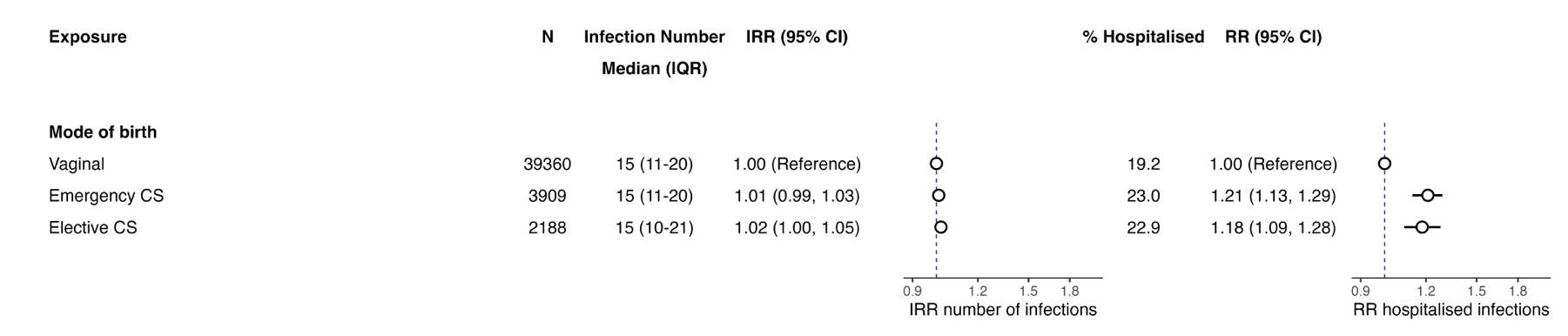 |
| --- | --- |
| **(B)** | 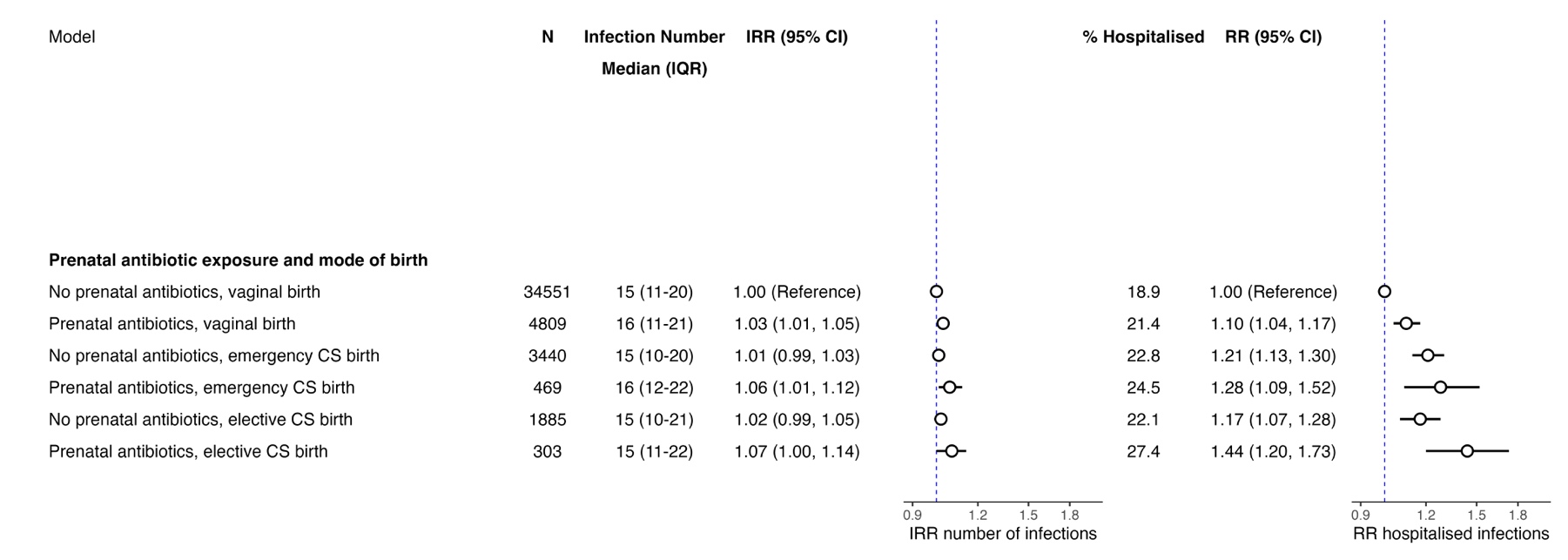 |
| **(C)** | 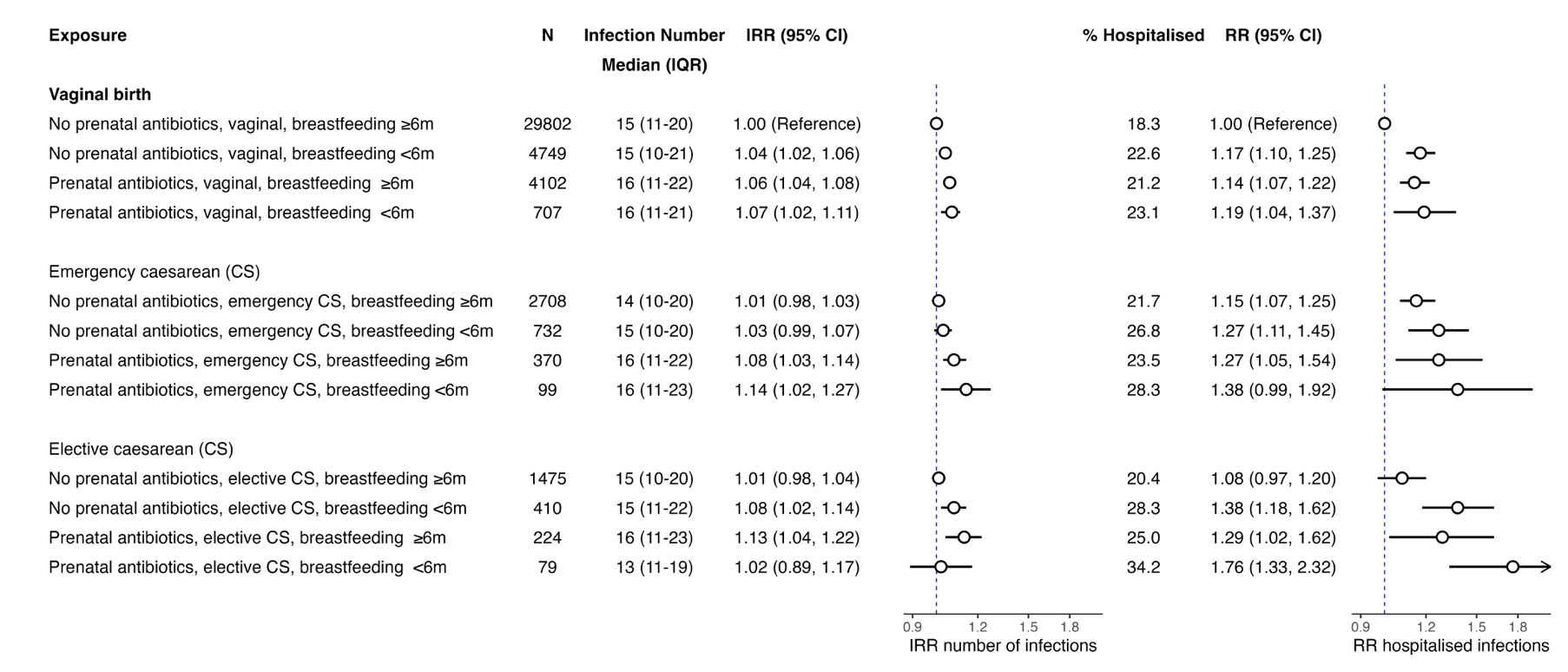 |
